# PTEN regulates adipose progenitor cell growth, differentiation, and replicative aging

**DOI:** 10.1016/j.jbc.2021.100968

**Published:** 2021-07-14

**Authors:** Anna S. Kirstein, Stephanie Kehr, Michèle Nebe, Martha Hanschkow, Lisa A.G. Barth, Judith Lorenz, Melanie Penke, Jana Breitfeld, Diana Le Duc, Kathrin Landgraf, Antje Körner, Peter Kovacs, Peter F. Stadler, Wieland Kiess, Antje Garten

**Affiliations:** 1 University Hospital for Children & Adolescents, Center for Pediatric Research, Leipzig University, Leipzig, Germany; 2 Bioinformatics Group, Department of Computer Science and Interdisciplinary Center for Bioinformatics, Leipzig University, Leipzig, Germany; 3 Medical Department III-Endocrinology, Nephrology, Rheumatology, Leipzig University Medical Center, Leipzig, Germany; 4 Institute of Human Genetics, Leipzig University Medical Center, Leipzig, Germany; 5 Department of Evolutionary Genetics, Max Planck Institute for Evolutionary Anthropology, Leipzig, Germany; 6 Max Planck Institute for Mathematics in the Sciences, Leipzig, Germany; 7 Institute for Metabolism and Systems Research, University of Birmingham, Birmingham, UK

**Keywords:** PTEN hamartoma tumor syndrome, adipogenesis, adipocyte, lipoma, cellular senescence, mesenchymal stem cells, FOXO1, forkhead box protein O1, KEGG, Kyoto Encyclopedia of Genes and Genomes, mTOR, mammalian target of rapamycin, NAMPT, nicotinamide phosphoribosyltransferase, pAKT, phosphorylated AKT, PHTS, PTEN hamartoma tumor syndrome, PI3K, phosphoinositide 3-kinase, pS6, ribosomal protein S6 phosphorylation, PTEN, phosphatase and tensin homolog, PTEN CR, PTEN CRISPR cells, PTEN KD, knockdown of PTEN, RNPs, ribonucleoproteins, SA-β-gal, senescence-associated β-galactosidase, SREBP1, sterol regulatory element-binding protein 1, SVF, stromal vascular fraction

## Abstract

The tumor suppressor phosphatase and tensin homolog (PTEN) negatively regulates the insulin signaling pathway. Germline PTEN pathogenic variants cause PTEN hamartoma tumor syndrome (PHTS), associated with lipoma development in children. Adipose progenitor cells (APCs) lose their capacity to differentiate into adipocytes during continuous culture, whereas APCs from lipomas of patients with PHTS retain their adipogenic potential over a prolonged period. It remains unclear which mechanisms trigger this aberrant adipose tissue growth. To investigate the role of PTEN in adipose tissue development, we performed functional assays and RNA-Seq of control and PTEN knockdown APCs. Reduction of PTEN levels using siRNA or CRISPR led to enhanced proliferation and differentiation of APCs. Forkhead box protein O1 (FOXO1) transcriptional activity is known to be regulated by insulin signaling, and FOXO1 was downregulated at the mRNA level while its inactivation through phosphorylation increased. FOXO1 phosphorylation initiates the expression of the lipogenesis-activating transcription factor sterol regulatory element-binding protein 1 (SREBP1). SREBP1 levels were higher after PTEN knockdown and may account for the observed enhanced adipogenesis. To validate this, we overexpressed constitutively active FOXO1 in PTEN CRISPR cells and found reduced adipogenesis, accompanied by SREBP1 downregulation. We observed that PTEN CRISPR cells showed less senescence compared with controls and the senescence marker CDKN1A (p21) was downregulated in PTEN knockdown cells. Cellular senescence was the most significantly enriched pathway found in RNA-Seq of PTEN knockdown *versus* control cells. These results provide evidence that PTEN is involved in the regulation of APC proliferation, differentiation, and senescence, thereby contributing to aberrant adipose tissue growth in patients with PHTS.

Adipose tissue distribution is altered during aging, while subcutaneous fat depots decrease in advanced age and fat accumulates in the muscle, liver, and bone marrow, leading to metabolic dysregulation (1). Responsiveness to insulin declines in adipocytes from older individuals and metabolic features related to fatty acid metabolism change (2, 3). Human adipose tissue contains mesenchymal stem cell, which serve as adipocyte progenitors and contribute to adipose tissue regeneration throughout life (4). These cells are found within the stromal vascular fraction (SVF) of adipose tissue. Adipogenesis can be induced by insulin and other soluble factors *in vitro* (5). SVF cells from older individuals have a lower capacity for adipocyte differentiation (6), and during long-term SVF cell culture, the adipogenic potential declines (7). Inhibiting the phosphoinositide 3-kinase (PI3K)/AKT pathway in adipose progenitors using the mammalian target of rapamycin (mTOR) inhibitor rapamycin (8) or the PI3K inhibitor alpelisib (9) was shown to repress adipogenesis. Several studies link insulin signaling and aging. Mice with adipose tissue–specific insulin receptor KO had an increased life span (10), but the underlying mechanisms are controversial (11). Adipose tissue in these mice maintains mitochondrial activity and insulin sensitivity during aging, indicating that insulin-sensitivity dynamics rather than insulin resistance correlate with longevity (11, 12).

We observed that lipoma cells from a patient with a phosphatase and tensin homolog (*PTEN*) germline pathogenic variant retain their differentiation capacity over a prolonged period (13). PTEN is a lipid and protein phosphatase, mainly catalyzing the dephosphorylation of the second messenger phosphatidylinositol-3,4,5-trisphosphate, resulting in a deactivation of the PI3K/AKT pathway signaling. PTEN is an important antagonist of this pathway, which is activated by a multitude of extracellular signals including insulin and insulin-like growth factor 1. PI3K/AKT signaling generally promotes cellular growth and survival. The downstream target and central signaling molecule mTOR is a major regulator of protein and lipid synthesis, cell growth, proliferation, autophagy, and metabolism (14). Loss of the tumor suppressor *PTEN* is common in cancer. *PTEN* haploinsufficiency caused by germline pathogenic variants leads to the rare genetic disease PTEN hamartoma tumor syndrome (PHTS). Patients with PHTS show a wide variety of phenotypes including hamartomas of the skin, breast, and thyroid, intestinal polyps, macrocephaly, vascular malformations, and lipoma formation (15). Widespread abdominal lipomatosis and lack of subcutaneous adipose tissue were observed in a boy with PHTS (16). It remains unclear which specific factors cause this localized adipose tissue overgrowth in patients with PHTS.

Several mouse models with *PTEN* downregulation in adipose tissue (17, 18), adipose progenitor subpopulations (17, 18), or osteoblast progenitors (19, 20) display adipose tissue redistribution and/or lipoma formation and partly recapitulate the human phenotype of PHTS. Overexpression of AKT in zebrafish also leads to lipoma formation, linking PI3K signaling to adipose tissue overgrowth (21). A high PTEN expression in adipose tissue (22) points to its importance in regulating normal adipose tissue function. *PTEN* pathogenic variants were found to lead to adipose tissue redistribution in mice (17, 18), with similar phenotypes also observed in humans (16).

To investigate the effects of PTEN downregulation in human adipose progenitor cells and create an *in vitro* model for PTEN insufficiency as seen in PHTS, we used SVF cells isolated from adipose tissue of healthy donors and downregulated PTEN *via* siRNA or CRISPR system. We thereby observed phenomena associated with proliferation, differentiation, and replicative aging of fat cell progenitors pointing to a role for PTEN in lipoma formation.

## Results

### PTEN downregulation enhanced PI3K signaling and SVF cell proliferation

**Figure 1:**
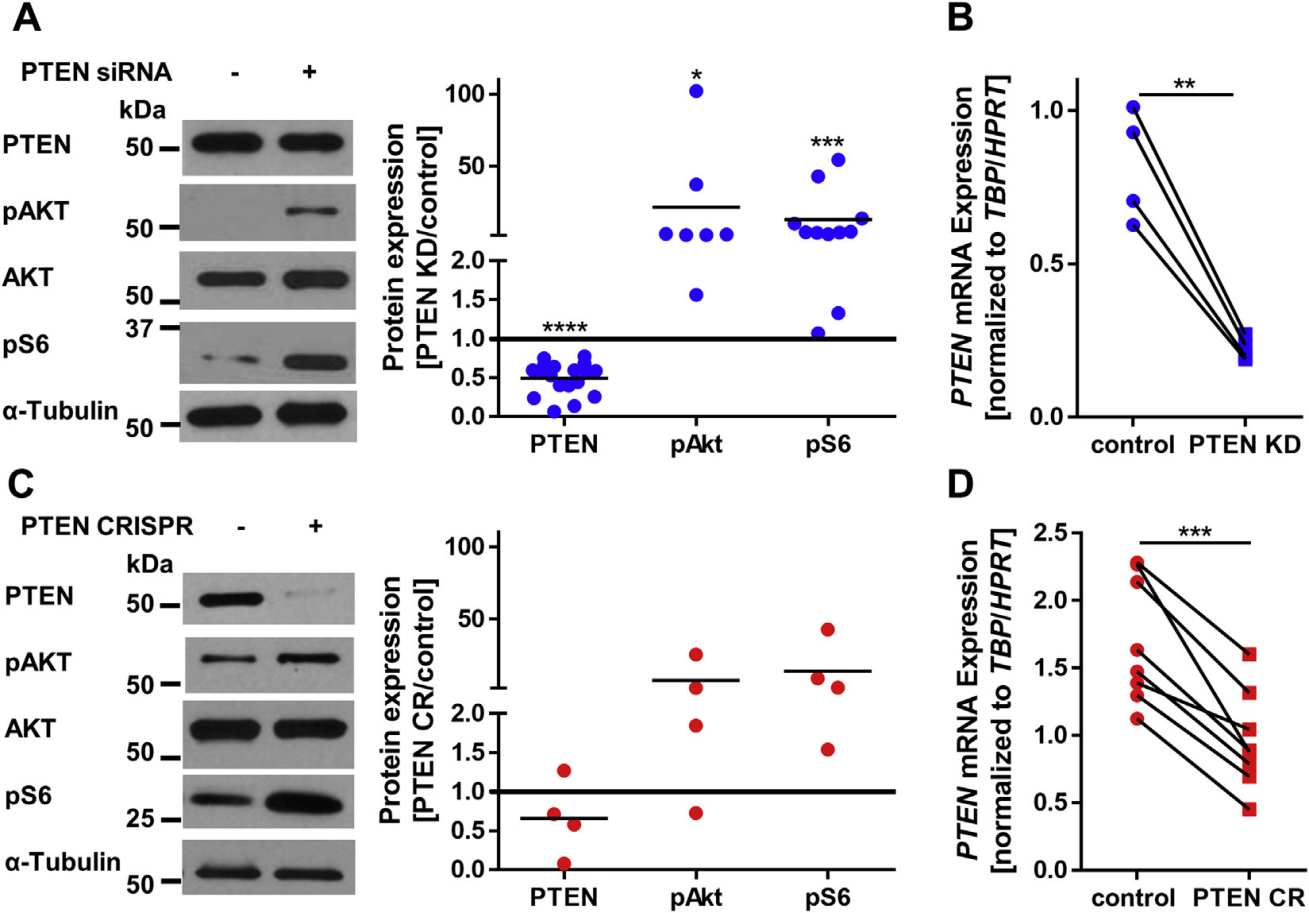
**PTEN downregulation enhances PI3K signaling.***A*, Western blots of control and PTEN siRNA (PTEN KD)-transfected visceral SVF cells: PTEN was reduced to 0.49 ± 0.05 fold (normalized to α-tubulin, n = 18, *p* < 0.0001), phosphorylated AKT (pAKT (T308)) was elevated 22 ± 14 fold (normalized to total AKT, n = 7, *p* = 0.029), and ribosomal protein S6 phosphorylation (pS6 (Ser235/236)) was increased 13.03 ± 5.5 fold (normalized to α-tubulin, n = 11, *p* = 0.0008). *B*, *PTEN* mRNA expression in control and PTEN siRNA-transfected visceral SVF cells: *PTEN* was reduced to 0.33 ± 0.08 fold (normalized to the means of hypoxanthine phosphoribosyltransferase (HPRT) and TATA-box–binding protein (TBP) expression, n = 4, *p* = 0.0039). *C*, Western blots of control and PTEN CRISPR (PTEN CR) SVF cells: PTEN was reduced to 0.7 ± 0.3 fold (normalized to α-tubulin, n = 4, *p* = 0.27), pAKT (T308) was increased 8 ± 6 fold (normalized to total AKT, n = 4, *p* = 0.24), and pS6 (Ser235/236) was increased 14 ± 10 fold (normalized to α-tubulin, n = 4, *p* = 0.09). *D*, *PTEN* mRNA expression in control and PTEN CR cells: *PTEN* was reduced to 0.56 ± 0.05 fold (*p* = 0.0002) (normalized to *HPRT* and *TBP*, n = 8, *p* = 0.0002). *Lines* between individual data points indicate matched data from single experiments (control *versus* respective knockdown). *p*-values for panels *A* and *C* were determined *via* one-sample *t* test of the log(fold change), and *p*-values for panels *B* and *D* were determined *via* paired *t* test (∗*p* < 0.05, ∗∗*p* < 0.01, ∗∗∗*p* < 0.001, ∗∗∗∗*p* < 0.0001). pAKT, phosphorylated AKT; PI3K, phosphoinositide 3-kinase; PTEN, phosphatase and tensin homolog; PTEN KD, knockdown of PTEN; SVF, stromal vascular fraction.

To examine the impact of PTEN loss on adipocyte development, we performed siRNA-mediated knockdown of PTEN (PTEN KD) in SVF cells from visceral and subcutaneous adipose tissue of donors without *PTEN* mutation. As determined *via* Western blot analysis, PTEN was reduced in the visceral siRNA KD cells to 0.49 ± 0.04 fold (*p* < 0.0001, Fig. 1*A*). On the mRNA level, we found a reduction of *PTEN* expression to 0.33 ± 0.08 fold (*p* < 0.0001) in visceral (Fig. 1*B*) and 0.3 ± 0.01 fold (*p* = 0.009) in subcutaneous SVF cells (Fig. S1*A*). The KD was stable during 7 days of proliferation (Fig. S1*B*) and 8 days of differentiation (Fig. S1*C*). To test the functional significance of the KD, we investigated the activation of the downstream PI3K/AKT pathway components *via* phosphorylation. Phosphorylated AKT (pAKT (T308)) was elevated 22 ± 14 fold (*p* = 0.029) and ribosomal protein S6 phosphorylation (pS6 (Ser235/236)) was increased 13.0 ± 5.5 fold (*p* = 0.0008) in visceral PTEN KD cells (Fig. 1*A*).

siRNA-mediated KDs are of transient nature, which is why we additionally performed *PTEN* KOs using the CRISPR system in visceral SVF cells (PTEN CR). On average, PTEN protein levels were reduced to 0.7 ± 0.3 fold (*p* = 0.27), while pAKT (T308) was increased 8 ± 6 fold (*p* = 0.24) and pS6 (Ser235/236) was increased 14 ± 10 fold (*p* = 0.09) (Fig. 1*C*). *PTEN* mRNA was reduced to 0.56 ± 0.05 fold (*p* = 0.0002) in PTEN CR cells (Fig. 1*D*). Using both methods, we achieved a similar reduction in PTEN levels, as seen in cells of a patient with germline heterozygous *PTEN* deletion (13).

**Figure 2:**
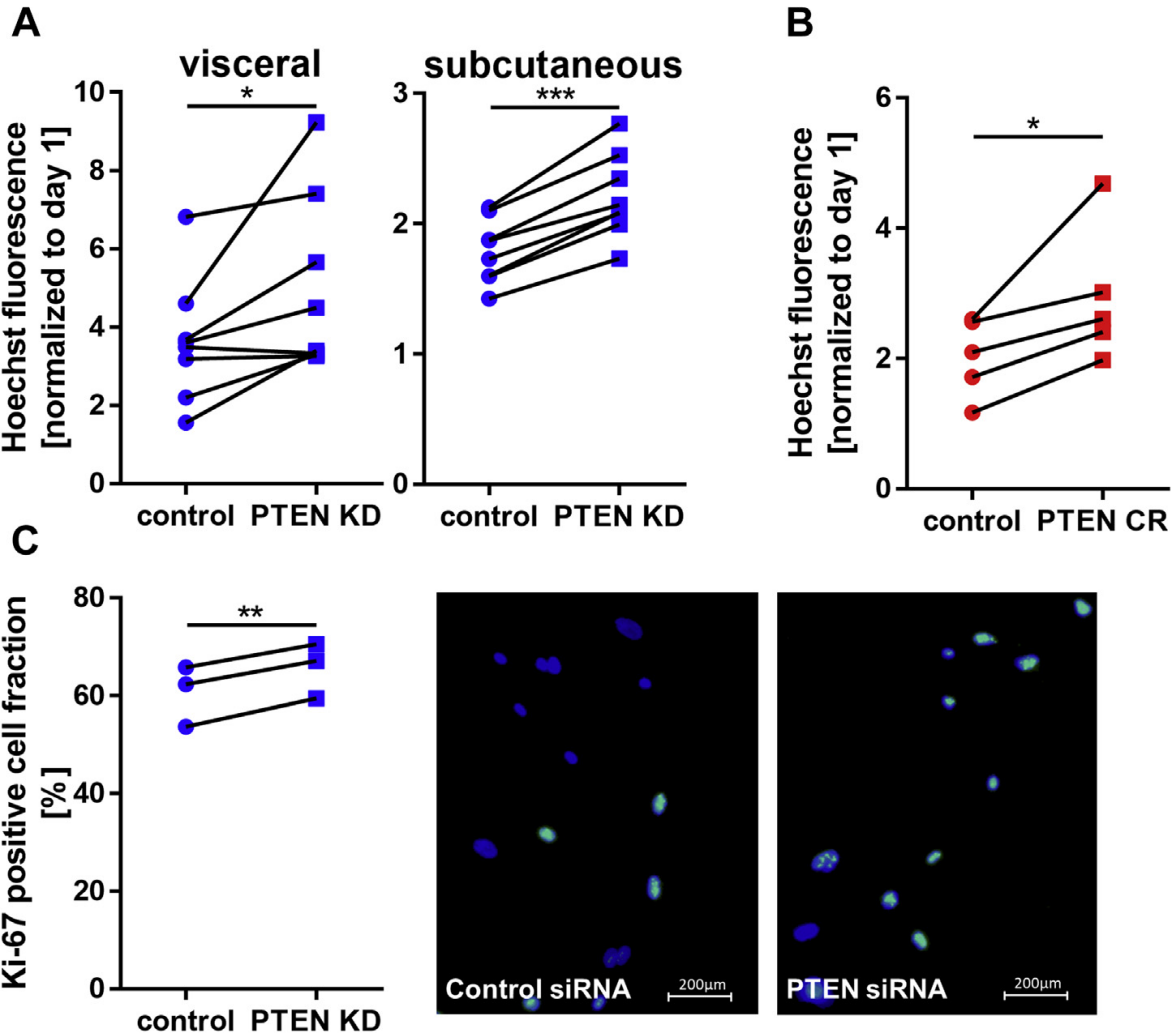
**PTEN downregulation enhances proliferation.***A*, Hoechst nuclei staining of control and PTEN KD cells 7 days after transfection: proliferation in PTEN KD cells was increased 1.4 ± 0.2 fold (n = 8, *p* = 0.038) in visceral and 1.23 ± 0.02 fold (n = 8, *p* < 0.0001) in subcutaneous SVF cells. *B*, Hoechst nuclei staining of the control and PTEN CR cells 7 days after plating: proliferation in PTEN CR cells was increased 1.5 ± 0.1 fold (n = 5, *p* = 0.039). *C*, Hoechst nuclei staining (*blue*) and Ki-67 immunofluorescence staining (*green*) in visceral control and PTEN KD cells 1 day after transfection: PTEN KD cells show 1.09 ± 0.01 fold (n = 3, *p* = 0.0043) higher fraction of proliferation marker Ki-67-positive cells. *Lines* between individual data points indicate matched data from single experiments (control *versus* respective knockdown). *p*-values were determined *via* paired *t* test (∗*p* < 0.05, ∗∗*p* < 0.01, ∗∗∗*p* < 0.001). PTEN, phosphatase and tensin homolog; PTEN CR, PTEN CRISPR cells; PTEN KD, knockdown of PTEN; SVF, stromal vascular fraction.

We observed an enhanced proliferation in PTEN-insufficient SVF cells. PTEN downregulation led to faster expansion to a similar extent in PTEN KD cells (1.4 ± 0.2 fold, *p* = 0.038 in visceral, 1.21 ± 0.08 fold, *p* = 0.0006 in subcutaneous SVF cells, Fig. 2*A*) and in PTEN CR cells (1.5 ± 0.1 fold, *p* = 0.039, Fig. 2*B*). The higher cell count was also reflected in a 1.09 ± 0.01 fold (*p* = 0.0043) higher fraction of proliferation marker Ki-67-positive cells as shown by immunofluorescence staining (Fig. 2*C*).

### PTEN downregulation restored adipogenic potential in high-passage SVF cells

**Figure 3:**
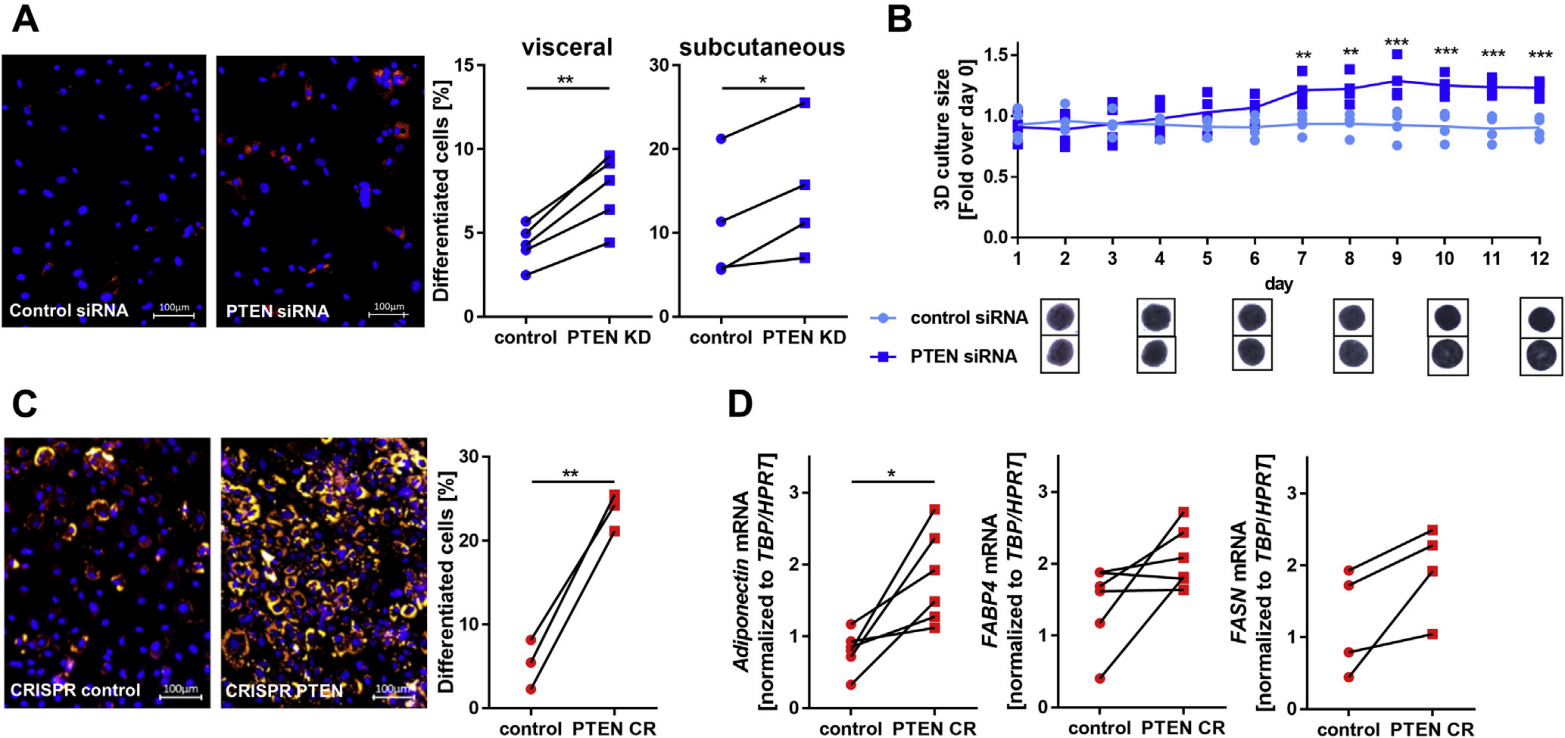
**PTEN downregulation enhances adipogenesis.***A*, Hoechst nuclei staining (*blue*) and Nile Red lipid staining (*red*) in high-passage SVF cells with or without PTEN KD after 8 days in the adipogenic medium: the fraction of differentiated cells increased 1.77 ± 0.07 fold (n = 5, *p* = 0.0026) in visceral and 1.44 ± 0.19 fold (n = 4, *p* = 0.0275) in subcutaneous SVF after PTEN knockdown. *B*, three-dimensional culture of visceral control and PTEN KD SVF cells in the adipogenic medium for 12 days: the size of control SVF cell spheroids remained constant over 12 days, while it was increased 1.23 ± 0.03 fold (n = 4, *p* < 0.001) for PTEN KD spheroids. *C*, Hoechst nuclei staining (*blue*) and Nile Red lipid staining (*red*) in high-passage control or PTEN CR SVF cells after 8 days in the adipogenic medium: The fraction of differentiated cells was increased 5.6 ± 1.9 fold (n = 3, *p* = 0.004) in CRISPR PTEN KO cells compared with controls. *D*, expression of the adipocyte markers in control or PTEN CR SVF cells after 8 days in the adipogenic medium: adiponectin expression was increased in PTEN CR cells 2.6 ± 0.6 fold (normalized to *HPRT* and *TBP*, n = 6, *p* = 0.016), fatty acid synthase (FASN) 2.1 ± 0.8 fold (normalized to *HPRT* and *TBP*, n = 4, *p* = 0.075), and fatty acid–binding protein 4 (FABP4) 1.9 ± 0.6 fold (normalized to *HPRT* and *TBP*, n = 6, *p* = 0.078) compared with controls. *Lines* between individual data points indicate matched data from single experiments (control *versus* respective knockdown). *p*-values for panels a, c, and d were determined *via* paired *t* test, and *p*-values for panels b were determined *via* one-way ANOVA followed by a post hoc Tukey's multiple comparisons test (∗*p* < 0.05, ∗∗*p* < 0.01, ∗∗∗*p* < 0.001). HPRT, hypoxanthine phosphoribosyltransferase; PTEN, phosphatase and tensin homolog; PTEN CR, PTEN CRISPR cells; PTEN KD, knockdown of PTEN; SVF, stromal vascular fraction; TBP, TATA-box–binding protein.

SVF cells lose their capacity for adipocyte differentiation when cultured for several passages (7). In view of the high adipogenic potential observed in PTEN haploinsufficient lipoma cells, we asked whether PTEN downregulation could reverse this process. High-passage SVF cells (>15 days in culture), which lost their adipocyte differentiation capacity, were transfected with *PTEN* or control siRNA and kept in adipogenic medium for 8 days. Nile Red lipid staining showed a 1.77 ± 0.07 fold (*p* = 0.0026) increase in visceral and 1.44 ± 0.19 fold (*p* = 0.0275) in subcutaneous SVF cells in differentiated cells after PTEN KD (Fig. 3*A*). This finding was also supported by an increase in size of three-dimensional spheroids of visceral PTEN KD SVF cells in the adipogenic medium (Fig. 3*B*). While the size of control siRNA spheroids remained constant over 12 days, the size of PTEN KD spheroids was increased 1.23 ± 0.03 fold (*p* < 0.001). It was previously shown that the size of the spheroids corresponds to the degree of differentiation (13, 23).

Taking advantage of the stable PTEN downregulation, we observed differentiation capacity of PTEN CR cells 2 to 6 weeks after transfection. After long-term culture, only 5 ± 2% of control cells differentiated into adipocytes during 8 days in the adipogenic medium, while 24 ± 1% of PTEN CR cells differentiated (Fig. 3*C*). This is a 5.6 ± 1.9 fold (*p* = 0.004) increase in differentiation in the PTEN CR cells. Expression of the adipocyte markers *adiponectin* (*ADIPOQ*) (2.6 ± 0.6 fold, *p* = 0.016), *fatty acid synthase* (*FASN*) (2.1 ± 0.8 fold, *p* = 0.075) and *fatty acid binding protein 4* (*FABP4*) (1.9 ± 0.6 fold, *p* = 0.078) was increased in PTEN CR cells compared with controls after 8 days in the adipogenic medium, confirming these findings (Fig. 3*D*).

### PTEN levels were upregulated during cellular aging

**Figure 4:**
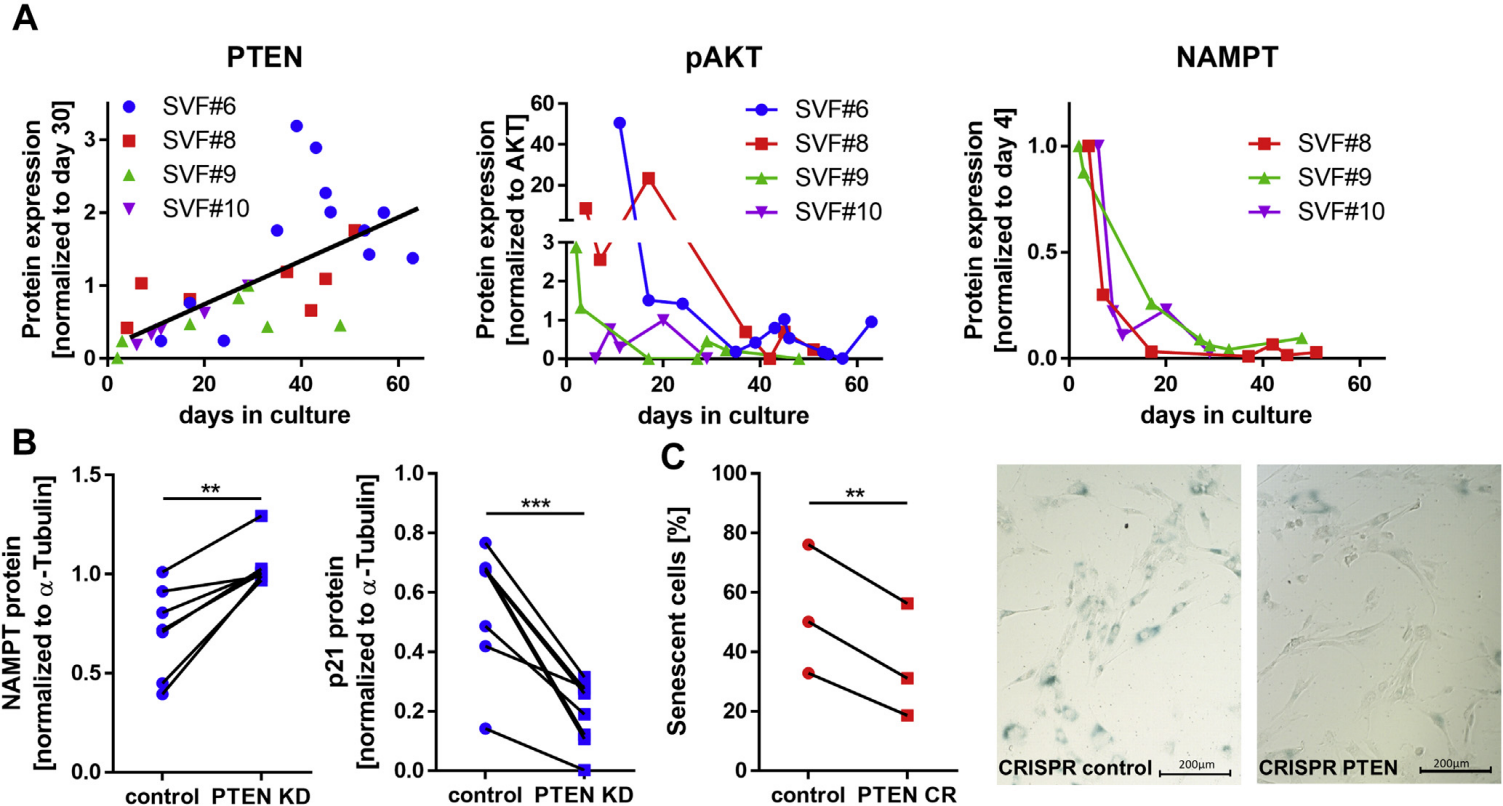
**PTEN levels increase during cellular aging.***A*, Western blots of primary visceral SVF cells at different days in culture: PTEN levels increased during long-term culture (normalized to α-tubulin, *p* < 0.0001), while the ratio of pAKT (T308) to total AKT (*p* = 0.1) and nicotinamide phosphoribosyltransferase (NAMPT, normalized to α-tubulin, *p* = 0.0004) decreased during long-term culture. *B*, Western blots of control and PTEN KD visceral SVF cells: NAMPT protein increased 1.6 ± 0.2 fold (normalized to α-Tubulin, n = 7, *p* = 0.0029), while p21 protein decreased to 0.33 ± 0.07 fold (normalized to α-tubulin, n = 8, *p* = 0.0004) in PTEN KD cells. *C*, senescence-associated β-galactosidase (SA-β-gal) assay of control and PTEN CR cells after long-term culture: the fraction of SA-β-gal-positive cells was 1.58 ± 0.12 fold (n = 3, *p* = 0.0098) increased in the controls. *Lines* between individual data points indicate matched data from single experiments (control *versus* respective knockdown). *p*-values for panels *A* were determined *via* linear regression and correlation analysis, *p*-values for panels *B* and *C* were determined *via* paired *t* test (∗∗*p* < 0.01, ∗∗∗*p* < 0.001). pAKT, phosphorylated AKT; PTEN, phosphatase and tensin homolog; PTEN CR, PTEN CRISPR cells; PTEN KD, knockdown of PTEN; SVF, stromal vascular fraction.

After the indication of restored differentiation capacity in high-passage SVF cells after PTEN downregulation, we investigated whether PTEN levels were regulated during long-term culture of SVF cells. We analyzed levels of PTEN protein and AKT phosphorylation (pAKT) in primary visceral SVF cells at different passages. In addition, we detected levels of the NAD biosynthesis enzyme nicotinamide phosphoribosyltransferase (NAMPT), which is known to be downregulated during aging (24). PTEN levels rose during long-term culture, whereas the ratio of pAKT (T308) to total AKT levels declined. A decline was also observed in the levels of NAMPT during long-term culture (Fig. 4*A*). We observed a reversion in visceral PTEN KD cells, with NAMPT protein 1.6 ± 0.2 fold (*p* = 0.0029) elevated in PTEN KD cells, whereas levels of the senescence-associated cell cycle regulator p21 protein were reduced to 0.33 ± 0.07 fold (*p* = 0.0004) after PTEN KD (Fig. 4*B*). We also found a reduction of *CDKN1A* (*p21*) (to 0.6 ± 0.06 fold, *p* = 0.031) and senescence marker *CDKN2A* (*p16*) (to 0.68 ± 0.05 fold, *p* = 0.014) mRNA in PTEN KD SVF cells (Fig. S2*A*). After long-term culture, there were 1.58 ± 0.12 fold (*p* = 0.0098) more senescence-associated β-galactosidase (SA-β-gal)-positive cells found in the controls than PTEN CR cells, supporting a rejuvenating effect of PTEN downregulation (Fig. 4*C*). We detected an upregulation of senescence markers *p21* and *HIPK2* but not *CDKN2B* (*p15*) in high-passage compared with low-passage cells from lipomas of patients with PHTS (LipPD1) (Fig. S2*B*).

### Expression changes after PTEN downregulation

**Figure 5:**
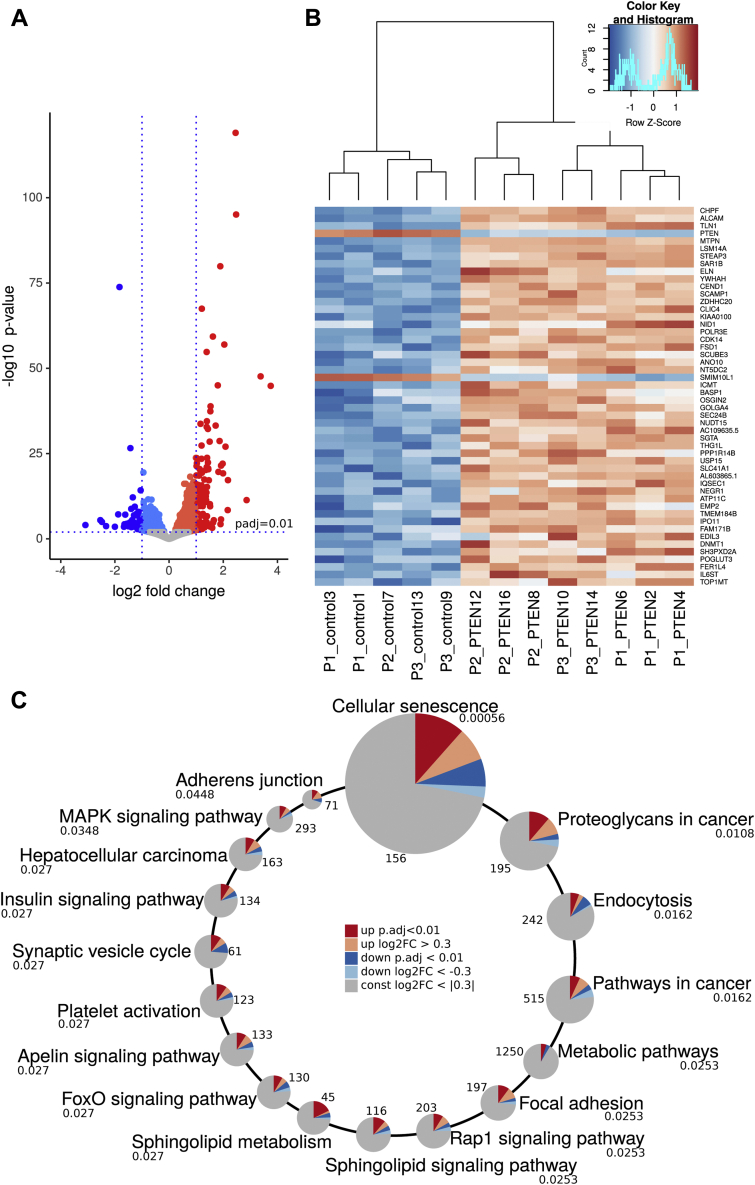
**RNA-Seq of PTEN knockdown and control visceral SVF cells.***A*, volcano plot of differential gene expression in control *versus* PTEN KD cells: we found 829 upregulated genes (*red*) and 550 downregulated genes (*blue*) (adjusted *p* < 0.01). Of those, 115 upregulated and 55 downregulated genes had a log2(fold change) (log2FC) >1 or <−1, which means at least duplication or halving of mRNA transcripts. *B*, the heatmap of differentially expressed genes in control *versus* PTEN KD cells: among the 50 most significantly differentially expressed genes, only *PTEN* and *SMIM10L1* were downregulated, whereas the majority of genes were upregulated. *C*, differentially expressed genes were significantly enriched (adjusted *p* < 0.05) in 18 KEGG pathways. The *circles* scale for adjusted *p*-values, which are given below the pathway name. *Numbers* next to the *circles* give the total number of genes assigned to the pathway in KEGG. *Dark red* and *blue slices* represent the fractions of significantly upregulated and downregulated genes in the pathways. *Light red* and *blue slices* mark additional fractions, where the expression is altered by at least 20% (log2FC >|0.3|), although the expression change was not significant. KEGG, Kyoto Encyclopedia of Genes and Genomes; PTEN, phosphatase and tensin homolog; PTEN KD, knockdown of PTEN; SVF, stromal vascular fraction.

We performed RNA-Seq of PTEN KD and control visceral SVF cells as an untargeted approach to identify genes and pathways regulated in conditions of PTEN downregulation (Tables S1 and S2 available at www.bioinf.uni-leipzig.de/publications/supplements/20-008). We found 1379 differentially expressed genes (adjusted *p*-value < 0.01), of which 170 had a log2(fold change) (log2FC) >1 or <−1 (Fig. 5*A*). More genes were upregulated 60% (829/1379) or 68% (115 of 170) than downregulated, corresponding to the pathway deactivating nature of PTEN (Fig. 5*B*). Among the 50 most significantly differentially expressed genes, only two were downregulated in PTEN KD (Fig. 5*B*). Gene set enrichment analysis using STRINGdb (25) identified 18 significantly enriched Kyoto Encyclopedia of Genes and Genomes (KEGG) pathways (*p* < 0.05) (Fig. 5*C*). The pathway most significantly enriched in PTEN KD compared with control SVF cells was cellular senescence (hsa04218) with altered gene expression levels for 28 of 156 genes (*p* = 0.00056). We could confirm the RNA-Seq results for senescence-associated genes *p15* and *HIPK2* *via* qPCR (Fig. S2*C*). Detailed networks of the regulated and other KEGG pathways relevant in the context of metabolism, aging, and adipocyte progenitor cells can be found at the supporting information website: www.bioinf.uni-leipzig.de/publications/supplements/20-008.

### Enhanced adipogenesis is mediated through forkhead box protein O1 (FOXO1) phosphorylation and sterol regulatory element-binding protein 1 (SREBP1)

**Figure 6:**
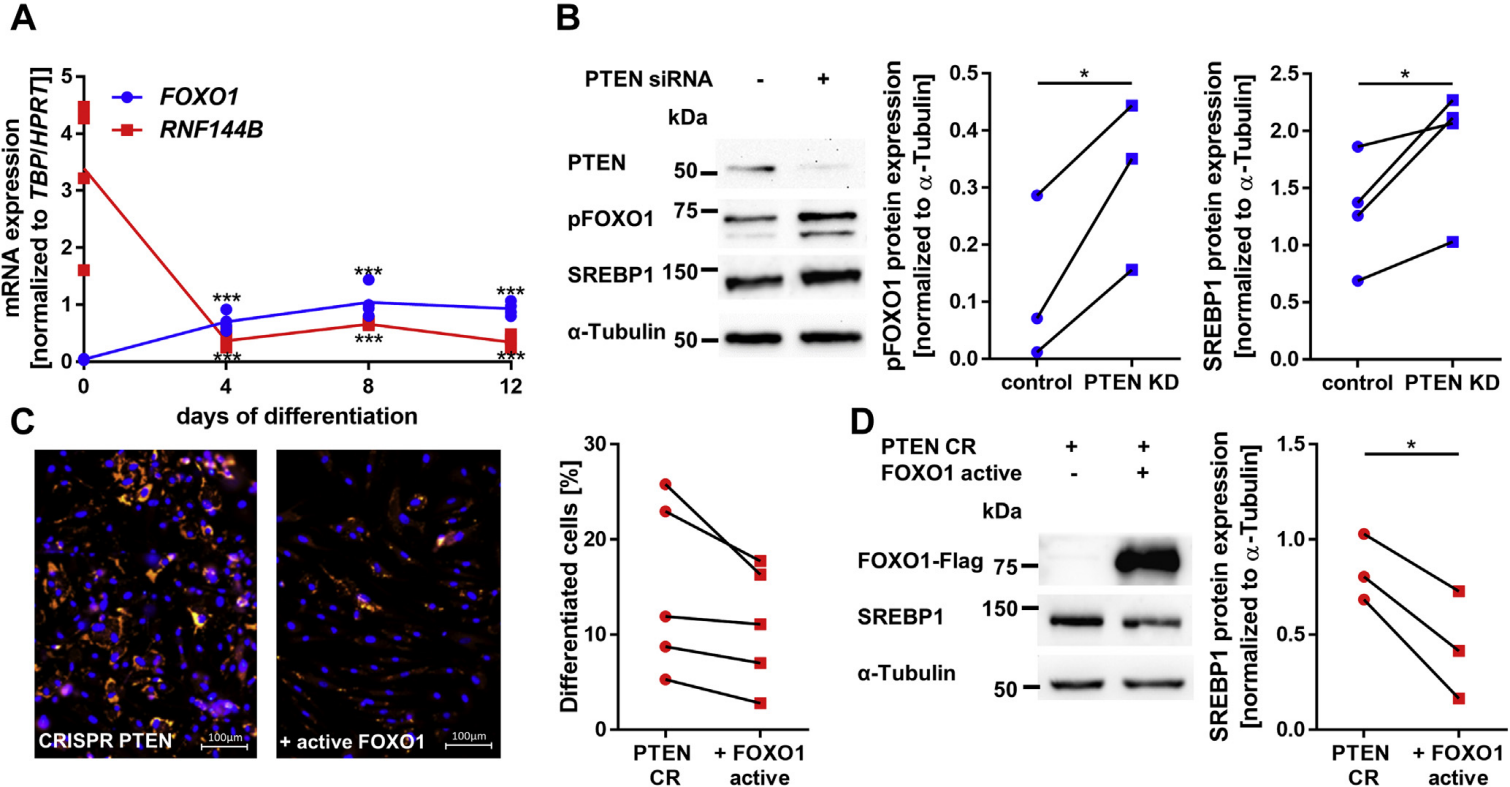
**PTEN knockdown induces SREBP1 expression *via* FOXO1 phosphorylation.***A*, gene expression of *FOXO1* and *RNF144B* during adipogenesis: *FOXO1* was upregulated during adipocyte development while *RNF144B* was downregulated (n = 4, ∗∗∗*p* < 0.001) in lipoma cells from a patient with PHTS. *B*, Western blots of phosphorylated FOXO1 (pFOXO1) and SREBP1 in control and PTEN KD visceral SVF cells: pFOXO1 was increased 6.6 ± 3.5 fold (n = 3, *p* = 0.046) and SREBP1 was increased 1.49 ± 0.13 fold (n = 4, *p* = 0.047) after PTEN KD. *C*, Hoechst nuclei staining (*blue*) and Nile Red lipid staining (*red*) in control and constitutively active FOXO1-overexpressing PTEN CR SVF cells after 8 days in the adipogenic medium: The fraction of differentiated cells decreased 0.73 ± 0.07 fold (n = 5, *p* = 0.065). *D*, Western blots of FOXO1 and SREBP1 in control and constitutively active FOXO1-overexpressing PTEN CR SVF cells: SREBP1 expression decreased 0.49 ± 0.14 fold (n = 3, *p* = 0.024) after FOXO1 overexpression. *Lines* between individual data points indicate matched data from single experiments (control *versus* respective knockdown/overexpression). *p*-values for panel *A* were determined *via* one-way ANOVA followed by a post hoc Tukey's multiple comparisons test, and *p*-values for panels *B*, *C*, and *D* were determined *via* paired *t* test (∗*p* < 0.05, ∗∗∗*p* < 0.001). PHTS, PTEN hamartoma tumor syndrome; PTEN, phosphatase and tensin homolog; PTEN KD, knockdown of PTEN; SVF, stromal vascular fraction.

To identify genes relevant for lipoma development, adipose tissue function, and adipogenesis, we compared our gene set with two other gene sets; first, a study by Le Duc *et al.* (26) comparing lipomas and subcutaneous adipose tissue of the same patient, and second, a study by Breitfeld *et al.* (27) comparing adipogenesis in murine inguinal and epididymal fat depots. We found an overlap of 36 genes (Table S3), including some known regulators of adipogenesis such as *FOXO1* (28) and genes such as *RNF144B*, which has no known function in adipose tissue yet. We confirmed the downregulation for these two genes *via* qPCR (Fig. S3*A*) and analyzed their expression changes during 12 days of adipogenesis in lipomas of patients with PHTS (LipPD1). We found *FOXO1* upregulated and *RNF144B* downregulated during adipocyte development (*p* < 0.001) (Fig. 6*A*). We also investigated the inactivation of FOXO1 through phosphorylation and found that pFOXO1 increased (6.6 ± 3.5 fold, *p* = 0.046) in PTEN KD cells (Fig. 6*B*). The FOXO1 downstream target SREBP1 was upregulated on the protein level (1.5 ± 0.1 fold, *p* = 0.047, Fig. 6*B*) and on the mRNA level (1.6 ± 0.1 fold, *p* = 0.013, Fig. S3*B*) in PTEN KD cells. To investigate whether a reintroduction of the downregulated FOXO1 and RNF144B could attenuate the effects of PTEN downregulation, we overexpressed constitutively active FOXO1 and RNF144B in PTEN CR cells. Although both had no consistent effect on proliferation of the PTEN CR cells (Fig. S3*C*), we observed an attenuating effect of constitutively active FOXO1 on adipogenesis (0.73 ± 0.07 fold, *p* = 0.065), whereas SREBP1 protein levels were reduced (0.49 ± 0.14 fold, *p* = 0.024). RNF144B overexpression had no influence on adipogenesis (Fig. S3*D*).

## Discussion

As a well-established tumor suppressor, PTEN is known for its antiproliferative effects in many cell types. However, not much is known about the role of PTEN in adipocyte progenitors. Both obesity and lipoma formation are characterized by adipose tissue overgrowth. Although it is known that in obesity both hypertrophy and hyperplasia contribute to adipose tissue expansion (29), it remains unclear whether these mechanisms also promote lipoma formation. Within this study, we investigated the mechanisms leading to aberrant adipose tissue growth and lipoma formation in patients with heterozygous *PTEN* mutations. A PTEN downregulation in adipocyte progenitor cells of approximately 50% as observed in lipoma cells of a patient with PHTS (13) leads to a several-fold activation of the PI3K downstream targets AKT and ribosomal protein S6. The overactivation of the PI3K pathway enhanced the proliferation of these cells, which is in line with the general cell cycle–activating function of the pathway (30). We conclude that PTEN controls adipose tissue growth and inhibits adipocyte progenitor expansion under normal conditions. Conversely, reduction in the phosphatase PTEN may lead to adipose tissue hyperplasia and lipoma formation.

We previously observed that SVF cells obtained from lipomas of pediatric patients with PHTS retain their ability to differentiate into adipocytes over a prolonged period, whereas primary SVF cells from healthy donors lose their differentiation capacity after several passages (13). To investigate whether this effect is caused by a reduction in PTEN protein levels, we analyzed differentiation of SVF cells with and without PTEN KD. SVF cells that had already lost their capacity to differentiate into adipocytes differentiated again in two-dimensional and three-dimensional culture after siRNA-mediated PTEN KD. SVF cells with stable CRISPR-mediated PTEN downregulation kept their ability to undergo differentiation after long-term culture. The SVF cells used within this study were obtained from obese donors, and it remains unclear whether this influences the effects seen after PTEN downregulation. We ensured to use SVF cells from nondiabetic donors to avoid altered insulin responses. Enhanced adipogenesis and proliferation was observed both in SVF cells from visceral and subcutaneous adipose tissue depots. In support of the hypothesis that PTEN plays a role in regulating adipogenesis, we identified several genes differentially expressed comparing PTEN KD and control cells. We validated two of these, *FOXO1* and *RNF144B*, showing their regulation during adipocyte differentiation. Because adipogenesis can be induced through insulin (5), the stimulating effects of PTEN downregulation on adipogenesis might be mediated through a higher insulin sensitivity. It is known that reduction in PTEN leads to higher insulin sensitivity, whereas the body weight increases (31), which could account for these effects. FOXO1 is phosphorylated through insulin signaling *via* AKT, inhibiting its transcriptional activity (32). We observed increased FOXO1 phosphorylation after PTEN KD and increased expression of the lipogenic transcription factor SREBP1, which is transcriptionally repressed by FOXO1 (33). Overexpression of constitutively active FOXO1 was accompanied by SREBP1 downregulation and led to attenuated adipogenesis in PTEN CRISPR cells. The inhibition of FOXO1 by AKT-mediated phosphorylation may account for the effects on adipogenesis observed after PTEN downregulation. The adipogenic potential of SVF cells from older animals is decreased (4). These age-related effects could be coupled to insulin sensitivity, which is also known to decrease with old age (1).

Interestingly, we found that PTEN protein levels are higher in SVF cells after several weeks in culture, connecting replicative age and adipogenic potential, which declines during long-term culture. A recent study of tissue-specific gene expression in mice during aging by Schaum *et al.* (34) showed a positive correlation of *PTEN* with age in subcutaneous fat depots, while most other tissues (including brown adipose tissue but not mesenteric adipose tissue) showed a negative correlation. These results indicate a relevance of PTEN in adipose tissue aging, not only *in vitro* but also *in vivo*. *p16* and *p21* expression as well as SA-β-gal expression were reported to be higher in SVF cells from older donors (35). To test whether there is a direct effect of PTEN on cellular senescence, we compared *p16* and *p21* expression with and without PTEN KD and found a reduction in KD cells. We observed less SA-β-gal-positive cells in SVF cells with CRISPR-mediated PTEN downregulation. These results provide evidence for a connection between PTEN expression and cellular senescence, although replicative aging processes are not completely blocked in PTEN haploinsufficient cells, as seen in comparison of senescence markers in low- and high-passage LipPD1 cells. In contrast to pathway activation through PTEN downregulation, PI3K inhibition with alpelisib induced senescence in PTEN haploinsufficient lipoma cells (9), and PTEN overexpression was shown to induce G1 arrest in cancer cells mediated through AKT inhibition (36). We found cellular senescence to be the most significantly enriched KEGG pathway in RNA-Seq of PTEN KD *versus* control SVF cells, with senescence-related genes such as *CDKN2B* (37) and *HIPK2* (38) downregulated. A relation between PI3K/AKT pathway and aging was also reported in other stem cell types: mTOR complex 1 activity in intestinal stem cells declines with aging (39). Age-related upregulation of the upstream antagonist PTEN might be responsible for this decline in downstream PI3K signaling pathway activity and could also account for the age-related decline in the mesenchymal stem cell proliferation rate (35). On the other hand, there is strong evidence that inhibition of the insulin signaling pathway with a special focus on mTOR complex 1 increases longevity. This makes sense in the context of insulin-induced metabolic activity and increased reactive oxygen species production (40). Some authors suggest an over proliferation and exhaustion of adipose progenitors in conditions of nutrient abundance associated with telomere shortening (41). Interestingly, Schumacher *et al.* (42) found similarities in differential gene expression between mouse models of delayed and premature aging compared with WT mice, with downregulation of insulin-like growth factor 1–related pathways as a common feature. Varying PTEN expression might be a cellular mechanism to maintain a balance between senescence and over proliferation, ensuring normal adipose tissue homeostasis. In disease states, PTEN downregulation leads to excessive adipose tissue growth and distribution abnormalities, whereas PTEN upregulation might contribute to senescence and dysfunctional adipose tissue in older individuals.

## Experimental procedures

### Cell culture and adipocyte differentiation

We used cells of the SVF isolated from visceral or subcutaneous adipose tissue of healthy donors, resected during bariatric surgery as well as PTEN-haploinsufficient lipoma cells (LipPD1) from a pediatric patient with PHTS (13). The study was approved by the Leipzig University ethics committee (ethical approval: no. 425-12-171220) and has been performed in accordance with the principles laid down in the 1964 Declaration of Helsinki and its later amendments. All adipose tissue and lipoma tissue donors gave written informed consent to participate in the study. Isolation and culture methods were described previously (16, 43). Table S4 contains a list of the primary cell cultures used. To characterize the cell populations found in these cultures, cell surface markers from SVF and LipPD1 cells cryopreserved at different passages were determined *via* flow cytometry analysis. Cells were thawed and resuspended in PBS supplemented with 0.5% BSA and 2 mM EDTA (pH 7.4). The cells were incubated 30 min at 4 °C, with fluorescently labeled monoclonal anti-human antibodies specific for CD8-PerCP, CD31-V450 (BD Biosciences), MSCA1-PE, CD271-APC, CD14-PeVio770, CD34-FITC (Miltenyi Biotec), and CD45-BV510 (BioLegend) (Table S5) (44). After washing steps, the cells were analyzed by flow cytometry using a FACS Aria II SORP flow cytometer and Diva Pro software (BD Biosciences) at the Core Unit Fluorescence Technologies of the University of Leipzig. Results from the analysis are presented in Table S6.

For differentiation, 15,000 cells/96-well or 120,000/12-well were plated in the culture medium. The medium was changed to differentiation medium 24 h later (day 0) (Dulbecco's modified Eagle's medium/F12 containing 8 mg/ml D-biotin, 10 mg/ml D-pantothenic acid, 2 μM rosiglitazone, 25 nM dexamethasone, 0.5 mM methylisobuthylxantine, 0.1 μM cortisol, 0.01 mg/ml apotransferrin, 0.2 nM triiodotyronin, and 20 nM human insulin (45)), and cells were kept in the differentiation medium for 4, 8, or 12 days.

A modified method according to Klingelhutz *et al.* (23) was used for scaffold-free three-dimensional cultures of SVF cells. After transfection, 10,000 cells per well were seeded into low attachment 96-well microplates (PS, U-bottom, clear, CELLSTAR, cell-repellent surface, Greiner Bio-One) in 100 μl culture medium. After 1 day, the medium was changed to the differentiation medium and spheroids were kept in the differentiation medium for 12 days. Half of the medium was replaced every second day. Microscope images were taken daily using the EVOS FL Auto 2 Cell Imaging System (Invitrogen; Thermo Fisher Scientific). Image analysis to determine the spheroid size was performed using ImageJ (46).

### PTEN siRNA transfection

One day before transfection, cells were plated at a density of about 1300 cells/cm^2^ to ensure optimal growth. For transfection, we used the Neon Transfection System 100 μl Kit (Invitrogen; Thermo Fisher Scientific, Inc). Cells were harvested *via* trypsinization and washed with DPBS. Either control siRNA (Silencer Negative Control No. 1 siRNA, Ambion, Thermo Fisher Scientific, Inc) or a combination of *PTEN* siRNA (s325 and s326, both Ambion, Thermo Fisher Scientific, Inc) was added to the cell pellets (final concentration of 10 nM in the culture medium after transfection). Pellets were resuspended in 100 μl R-buffer for transfection and electroporated in Neon 100 μl tips at 1300 V, 2 pulses, 20 ms. After electroporation, cells were transferred to a prewarmed culture medium and distributed to multiwell tissue culture plates for functional assays.

### CRISPR/Cas *PTEN* KO

For stable KO of *PTEN* in SVF cells, we used the CRISPR/Cas9 genome-editing technique in a reverse transfection of guide RNA/Cas9 nickase ribonucleoproteins (RNPs). Cas9 nickase was chosen to avoid off-target effects. If not otherwise stated, reagents were purchased from Integrated DNA Technologies (Alt-R CRISPR-Cas9 system). Transfections were performed according to the manufacturers’ protocol with a combination of two different crRNA:tracrRNA guides and Alt-R S.p. Cas9 D10A Nickase 3 NLS (# 1078729). We tested combinations of four different crRNAs (Table S7) for their editing efficiency *via* the T7EI assay (# 1075931, Alt-R Genome Editing Detection Kit). For further assays, we used the most efficient guide combination (#3 and #4). RNPs of Cas9 nickase and Alt-R CRISPR-Cas9 Negative Control crRNA #1 (#1072544) were used for controls. Ten thousand SVF cells per well were transfected in a 48-well format with a final concentration of 10 nM crRNA:tracrRNA:Cas9 nickase RNPs. We used a total volume of 300 μl per well with 2.4 μl Lipofectamine RNAiMAX (#13778075, Thermo Fisher Scientific). Efficiency was determined 2 days after transfection *via* the T7EI assay.

### FOXO1 and RNF144B overexpression

Constitutively active FOXO1 and RNF144B were overexpressed in PTEN CR cells. One day before transfection, cells were plated at a density of about 1300 cells/cm^2^ to ensure optimal growth. For transfection, we used the Neon Transfection System 100 μl Kit (Invitrogen; Thermo Fisher Scientific, Inc). Cells were harvested *via* trypsinization and washed with DPBS. A control vector (c-Flag pcDNA3 was a gift from Stephen Smale [Addgene plasmid #20011; http://n2t.net/addgene:20011;RRID:Addgene_20011 (47)]), a vector for overexpression of constitutively active FOXO1 with mutated phosphorylation site (pcDNA3 Flag FKHR AAA mutant was a gift from Kunliang Guan [Addgene plasmid #13508; http://n2t.net/addgene:13508;RRID:Addgene_13508] (32)) or a vector for RNF144B overexpression (RNF144B pcDNA3.1+ C-(K)-DYK #OHu07981D, GenScript) was added to the cell pellets (final concentration of 1 μg/ml for FOXO1 and 2 μg/ml for RNF144B in the culture medium after transfection). Pellets were resuspended in 100-μl R-buffer for transfection and electroporated in Neon 100 μl tips at 1300 V, 2 pulses, 20 ms. After electroporation, cells were transferred to the prewarmed culture medium and distributed to multiwell tissue culture plates for functional assays.

### Lipid staining

For lipid staining, cells were fixed in 4% paraformaldehyde and washed with DPBS. Afterward, cells were costained with the fluorescent dyes Nile Red (0.5 μg/ml, Sigma) and Hoechst 33342 (1 μg/ml, Sigma) for 5 min in DPBS. Microscope images were taken with the EVOS FL Auto 2 Cell Imaging System (Invitrogen; Thermo Fisher Scientific), and cell counting was performed with the Celleste Image Analysis Software (Thermo Fisher Scientific).

### Proliferation assay

For proliferation assays, cells were seeded at a density of 2000 cells/well on 96-well plates. The growth medium was replaced every 72 h. Cells were fixed at day 1 and day 7 after transfection and kept in DPBS at 4 °C after fixation. Hoechst 33342 (Sigma) was used to stain nuclei for 5 min at a concentration of 1 μg/ml in DPBS. Hoechst fluorescence was detected at 455 nm and compared for day 1 and day 7.

### Western blot analysis

For Western blot analysis, transfected cells were seeded at a density of 10,000 cells/cm^2^ in the culture medium. The cell culture medium was replaced by the serum-free medium 1 day after transfection, and cells were harvested on the next day. For Western blot analysis of long-term cultures, cells were trypsinized during normal culture and 100,000 cells were frozen as a pellet. Proteins were extracted and immunoblotting was performed as described elsewhere (16). We used 10 μg protein per lane and incubated with primary antibodies (Cell Signaling Technology) and secondary antibodies (Dako; Agilent Technologies) according to Table S8. α-Tubulin was used as the loading control. Densitometric analysis was performed using ImageJ (46), and images of exposed films and their analysis are provided in Fig. S4 (PTEN KD), Fig. S5 (PTEN CR), and Fig. S6 (long-term culture).

### Immunofluorescence staining

For Ki-67 immunofluorescence staining, transfected cells were seeded at a density of 2000 cells/well on 96-well plates and fixed with 4% paraformaldehyde after 24 h. Cells were permeabilized and blocked in IF buffer (DPBS + 5% BSA +0.3% Tween 20) for 1 h at room temperature (RT) and stained with Ki-67 primary antibody overnight at 4 °C (Table S8). Cells were washed three times for 5 min with IF buffer and incubated with secondary Alexa Fluor 488 antibody (Table S8) for 2 h at RT in the dark. Afterward, cells were washed one time with IF buffer, then incubated 5 min with 1 μg/ml Hoechst 33342 in DPBS, and washed one time with DPBS. Microscope images were taken in DPBS using the EVOS FL Auto 2 Cell Imaging System (Invitrogen; Thermo Fisher Scientific), and cell counting was performed with the Celleste Image Analysis Software (Thermo Fisher Scientific).

### RT-qPCR

We plated 6000 cells/cm^2^ for RT-qPCR of undifferentiated cells or 45,000 cells/cm^2^ for differentiated cells. RNA extraction, reverse transcription, and qPCR were performed as previously described (43). Table S9 contains a list of primers used for qPCR assays. Results were normalized to the housekeepers hypoxanthine phosphoribosyltransferase and TATA box–binding protein. We performed probe-based assays using the Takyon Low Rox Probe MasterMix dTTP Blue (Eurogentec) or SYBR green assays using the Takyon Low Rox SYBR MasterMix dTTP Blue (Eurogentec).

### RNA-Seq

After PTEN KD, we plated 6000 cells/cm^2^ in the culture medium. The medium was replaced by the serum-free medium 1 day after transfection and cells were harvested 24 h afterward. RNA was extracted as for RT-qPCRs. 100 ng of total RNA was used for library synthesis with the NEBNext Ultra II Directional RNA Library Preparation kit (New England Biolabs) according to the protocol of the manufacturer. The barcoded libraries were purified and quantified using the Library Quantification Kit—Illumina/Universal (KAPA Biosystems) on a TaqMan 7500 Real-Time PCR System. A pool of up to ten libraries was used for cluster generation at a concentration of 10 nM. Sequencing of 2 × 150 bp was performed with a NextSeq 550 sequencer (Illumina) at the sequencing core facility of the Faculty of Medicine (Leipzig University) according to the instructions of the manufacturer.

#### Read preprocessing and mapping

Sequencing reads from 18 fastq files (triplicates of PTEN KD and controls from three donors) were mapped to the human reference genome (hg38 downloaded from UCSC/Ensembl (https://genome.ucsc.edu/index.html)). Paired-end RNA-Seq data were processed within the Galaxy platform (48). The raw sequencing reads (in fastq format) were loaded to Galaxy instance, and the quality was inspected with fastqc (https://www.bioinformatics.babraham.ac.uk/projects/fastqc/). One sample (PTEN KD and the respective control) had to be removed from further analysis because of low quality. Reads were trimmed with Trim Galore! (http://www.bioinformatics.babraham.ac.uk/projects/trim_galore/), and sufficient quality was ensured by a second quality check with fastqc. Subsequently, the reads were mapped to the human genome (hg38) with segemehl (49) and annotated with gencode.v29.

#### Differential gene expression and gene set enrichment

For differential gene expression analysis with DESeq2 (50), only genes with at least ten mapped reads in at least half of the samples were kept, reducing the analysis from 58,721 to 32,660 genes. After inspection of *PTEN* expression levels in all samples, we decided to exclude controls with only moderate *PTEN* expression levels to ensure clear distinguishability between high *PTEN* expression levels and *PTEN* KD. Thus, controls 5, 11, and 15 were excluded from further analyses (Fig. S7*A*). Afterward, we ran DESeq2 to identify differentially expressed genes. A principal component analysis identified the different donors are the main source of variance; thus, we corrected for the donor (batch) effect (Fig. S5*B*), leading to better clustering of PTEN KD *versus* control. We applied log2 fold change shrinkage to reduce effect sizes of overall lowly expressed genes (shrinkage estimator ashr) (51).

Gene set enrichment analysis was performed in STRING (string-db.org) (25). As input, the 1379 significantly differentially expressed genes were used. The results were visualized using R custom scripts and the KEGG pathway visualization package pathview (52).

### Statistical analysis

The means of at least three independent experiments were statistically analyzed using GraphPad Prism 6 software (GraphPad Software Inc). For comparison of control and conditions of PTEN downregulation (PTEN KD or PTEN CR), means of independent experiments were compared *via* paired Student's *t* test (comparison of each control transfection with the respective PTEN siRNA/PTEN CRISPR transfection). For multiple comparisons, we used one- or two-way ANOVA followed by a post hoc Tukey's multiple comparisons test (one-way ANOVA) or Dunnett's multiple comparisons test (two-way ANOVA). To determine the significance of fold changes, we used one-sample t-tests of the log(fold change) and compared with the hypothetical value 0 (53). Results are indicated as the mean ± SEM.

## Data availability

All data are contained within the article and in the supporting information. Gene lists and other supporting information are also available at http://www.bioinf.uni-leipzig.de/publications/supplements/20-008.

## Supporting information

This article contains supporting information.

## Conflict of interest

The authors declare that they have no conflicts of interest with the contents of this article.
