# Supplementary material for: PTEN regulates adipose progenitor cell growth, differentiation, and replicative aging
Source: J Biol Chem. 2021 Jul 14;297(2):100968. doi: 10.1016/j.jbc.2021.100968 (PMC8350019; doi:10.1016/j.jbc.2021.100968)
Supplement: Supplemental Figures S1–S7 and Tables S4–S9 [file mmc1.pdf]

Supporting information

Supporting information figures:

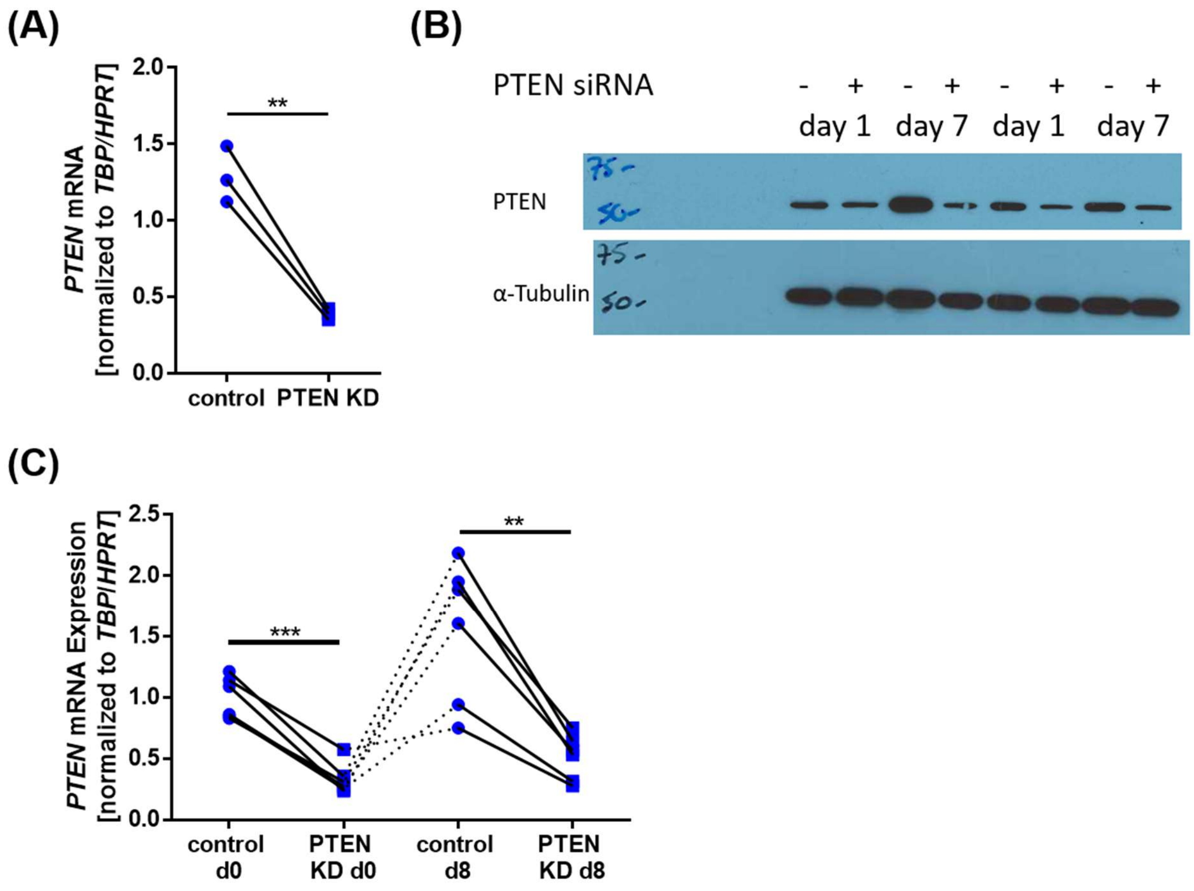

Figure S1: PTEN expression was reduced in control and PTEN KD SVF cells. (A) PTEN mRNA expression in subcutaneous PTEN KD SVF cells was downregulated 0.3 ± 0.01 fold (n=3, p=0.009) compared to controls. (B) Western blots of visceral PTEN KD/control SVF cells: PTEN knockdown was stable for 7 days after siRNA transfection. (C) *PTEN* mRNA expression in visceral PTEN KD/ SVF cells was downregulated upon induction of differentiation (d0, one day after transfection) and after eight days of adipose differentiation (d8) compared to controls. Lines between individual data points indicate matched data from single experiments (control vs. respective knockdown). p-values were determined via paired t-test (** p<0.001 *** p<0.001).

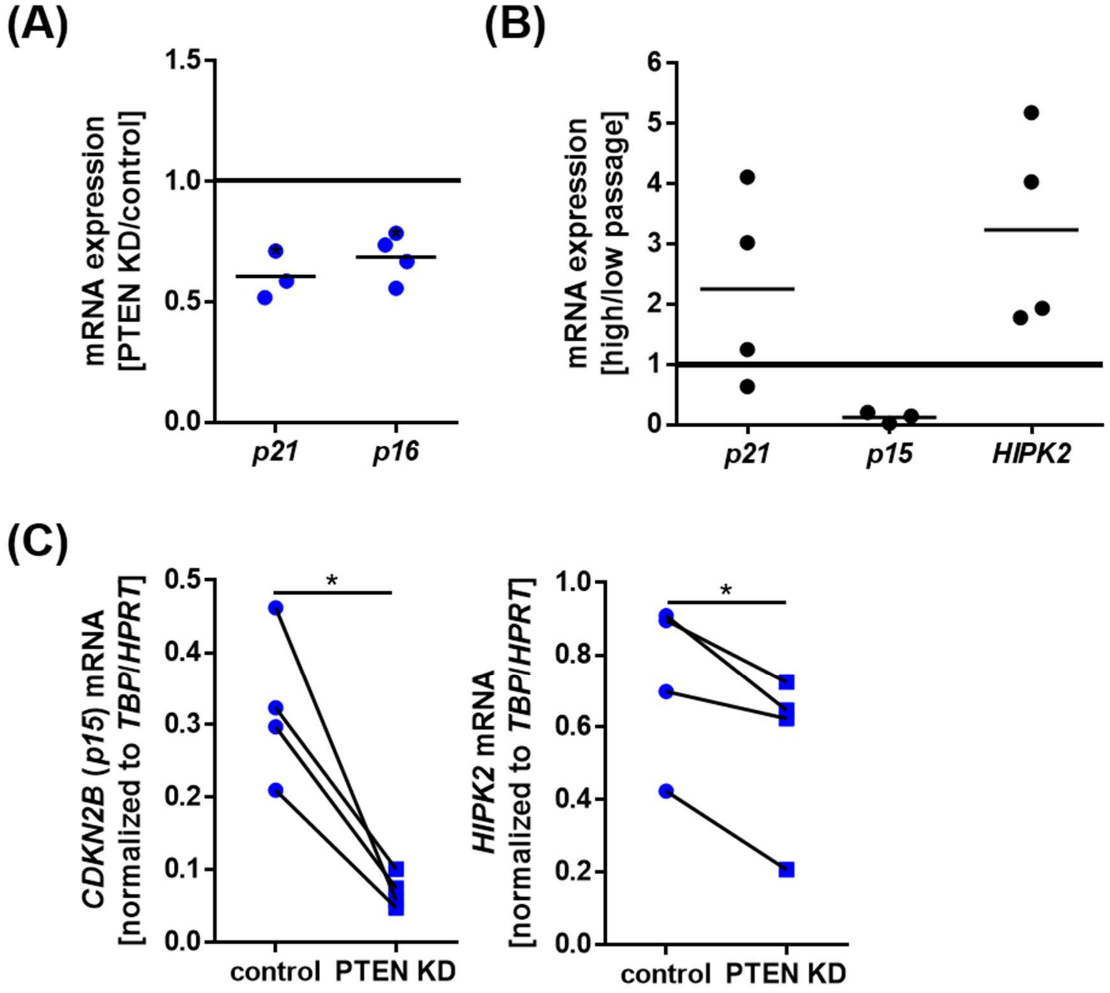

Figure S2: mRNA expression of senescence marker was reduced after PTEN KD. (A) Senescence marker qPCRs of PTEN KD/control visceral SVF cells: On the mRNA level we found a reduction of *CDKN1A* (*p21*) (to 0.6 ± 0.06 fold, n=3, p=0.031) and senescence marker *CDKN2A* (*p16*) (to 0.68 ± 0.05 fold, n=4, p=0.014). (B) Senescence marker qPCRs comparing low passage vs. high passage LipPD1 cells: On the mRNA level we found *p21* elevated 2.3 ± 0.8 fold, (n=4, p=0.27), *CDKN2B* (*p15*) reduced to 0.02 ± 0.05 fold (n=3, p=0.07) and HIPK2 elevated 3.2 ± 0.8 fold (n=4, p=0,028). (C) qPCRs to confirm results from RNA-sequencing: *p15* (to 0.23 ± 0.04 fold, n=4, p=0.017) and *HIPK2* (to 0.73 ± 0.09 fold, n=4, p=0.02) were downregulated after PTEN KD in visceral SVF cells. Lines between individual data points indicate matched data from single experiments (control vs. respective knockdown). p-values for (A) and (B) were determined via one-sample t-test of the log(fold change), p-values for (C) were determined via paired t-test.

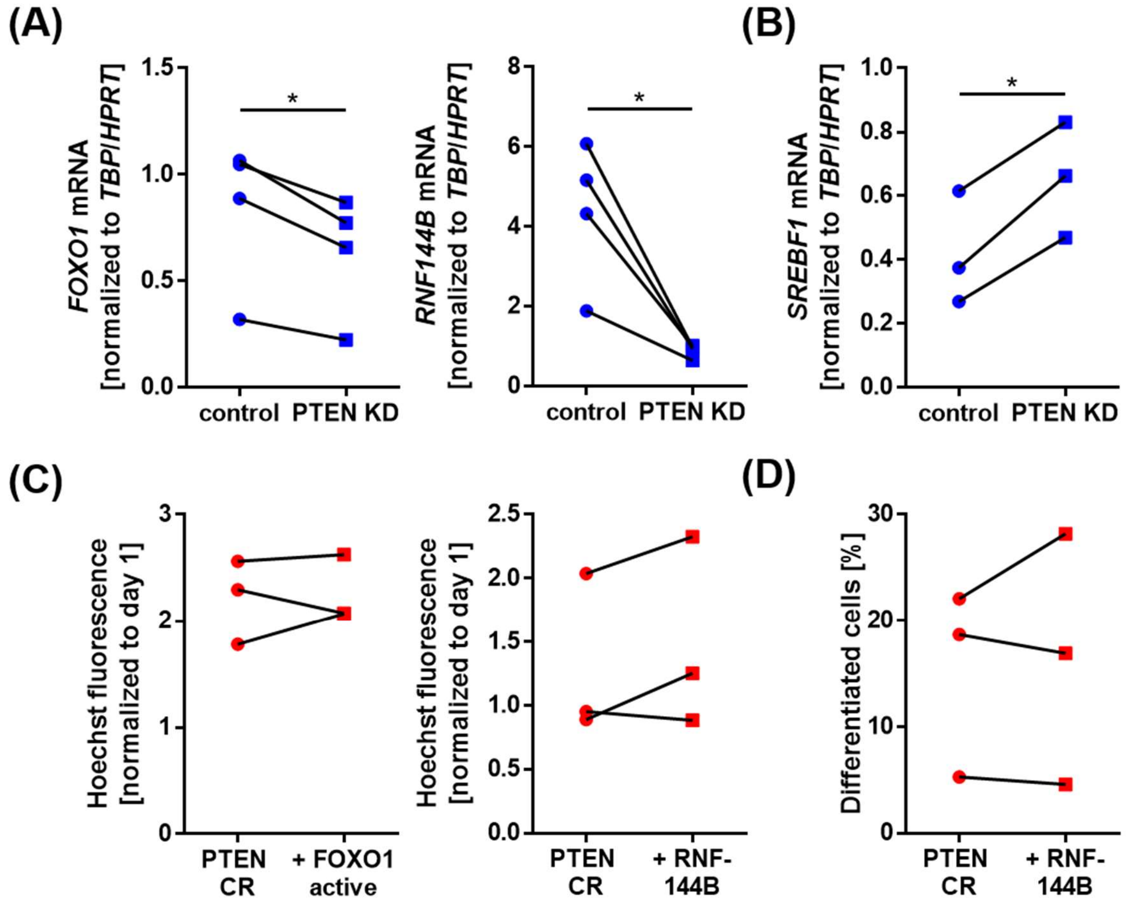

Figure S3: FOXO1 and RNF144B are downregulated in PTEN KD cells. (A) qPCRs to confirm results from RNA-sequencing: *FOXO1* (to 0.75 ± 0.03 fold, n=4, p=0.018) and *RNF144B* (to 0.23 ± 0.04 fold, n=4, p=0.025) were downregulated after PTEN KD in visceral SVF cells. (B) *SREBF1* was upregulated on the mRNA level (1.6 ± 0.1 fold, n=3, p=0.013) in visceral PTEN KD cells. (C) Neither overexpression of constitutively active FOXO1 nor RNF144B in PTEN CR cells had effects on proliferation (as determined via Hoechst assay 7 days post transfection normalized to day 1 after transfection). (D) RNF144B overexpression had no influence on adipogenesis of PTEN CR cells. Lines between individual data points indicate matched data from single experiments (control vs. respective knockdown or overexpression). p-values were determined via paired t-test.

PTEN/α-Tubulin

Gel 1

Lane	cell strain	passage	generation	siRNA	α-Tubulin	PTEN	PTEN/Tubulin	Fold
1	SVF#10	15	17.5	control	29685.8	30858.8	1.0395	1
2	SVF#10	15	17.5	PTEN	25892.3	6868.3	0.2653	0.255
3	SVF#10	19	20.5	control	26662.7	25792.9	0.9674	1
4	SVF#10	19	20.5	PTEN	25048.1	14121.5	0.5638	0.583
5	SVF#10	21	21.5	control	21221.5	16851.7	0.7941	1
6	SVF#10	21	21.5	PTEN	24097.4	1199.8	0.0498	0.063

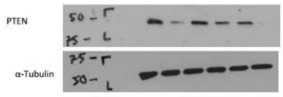

Gel 2

Lane	cell strain	passage	generation	siRNA	α-Tubulin	PTEN	PTEN/Tubulin	Fold
1	SVF#6	20	19.5	control	28432.6	34222.4	1.2036	1
2	SVF#6	20	19.5	PTEN	28163.5	13651.6	0.4847	0.403
3	SVF#6	23	25	control	28809.4	23717.6	0.8233	1
4	SVF#6	23	25	PTEN	26735.5	13076.3	0.4891	0.594
5	SVF#6	13	15	control	32007.1	36336.6	1.1353	1
6	SVF#6	13	15	PTEN	27552.4	13760.5	0.4994	0.44

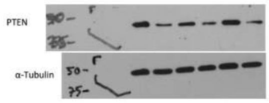

Gel 3

Lane	cell strain	passage	generation	siRNA	α-Tubulin	PTEN	PTEN/Tubulin	Fold
1	SVF#9	12	14.5	control	26326.2	34254.7	1.3012	1
2	SVF#9	12	14.5	PTEN	23683.8	18228.0	0.7696	0.592
3	SVF#9	16	19.5	control	26977.9	38985.1	1.4451	1
4	SVF#9	16	19.5	PTEN	23271.4	4514.9	0.1940	0.134
5	SVF#9	19	23	control	22783.1	25602.5	1.1237	1
6	SVF#9	19	23	PTEN	25241.9	6661.7	0.2639	0.235

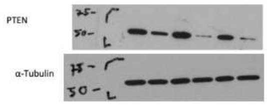

Gel 4

Lane	cell strain	passage	generation	siRNA	α-Tubulin	PTEN	PTEN/Tubulin	Fold
1	SVF#10	18	27	control	25150.6	20024.3	0.7962	1
2	SVF#10	18	27	PTEN	29801.7	17714.1	0.5944	0.747
3	SVF#9	25	29.5	control	28390.2	24607.5	0.8668	1
4	SVF#9	25	29.5	PTEN	31141.2	14283.6	0.4587	0.529
5	SVF#10	16	24.5	control	42170.0	53220.4	1.2620	1
6	SVF#10	16	24.5	PTEN	49634.0	41527.7	0.8367	0.663
7	SVF#10	19	28	control	36816.0	36749.9	0.9982	1
8	SVF#10	19	28	PTEN	33632.1	18851.5	0.5605	0.562

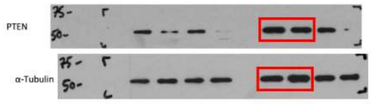

Gel 5

Lane	cell strain	passage	generation	siRNA	α-Tubulin	PTEN	PTEN/Tubulin	Fold
1	SVF#10	22	31.5	control	25567.1	26008.5	1.0173	1
2	SVF#10	22	31.5	PTEN	22026.5	14362.9	0.6521	0.641
3	SVF#8	6	11.5	control	23997.8	27608.4	1.1505	1
4	SVF#8	6	11.5	PTEN	22733.3	20224.4	0.8896	0.773
5	SVF#6	19	27.5	control	25526.2	28333.1	1.1100	1
6	SVF#6	19	27.5	PTEN	22336.8	14500.3	0.6492	0.585
7	SVF#6	10	16.5	control	32681.5	48733.2	1.4912	1
8	SVF#6	10	16.5	PTEN	31490.7	31924.4	1.0138	0.68
9	SVF#6	14	21	control	23326.8	21936.5	0.9404	1
10	SVF#6	14	21	PTEN	27925.7	10446.5	0.3741	0.398

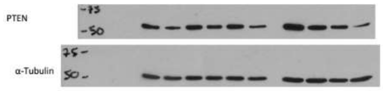

pAKT/AKT

Gel 1

Lane	cell strain	passage	generation	siRNA	pAKT	AKT	pAKT/AKT	Fold
1	SVF#10	22	31.5	control	8394.953	24410.3	0.3439	1
2	SVF#10	22	31.5	PTEN	22735.38	28621.7	0.7943	2.31
3	SVF#8	6	11.5	control	412.92	27179.5	0.0152	1
4	SVF#8	6	11.5	PTEN	915.749	26966.7	0.0340	2.235
5	SVF#6	19	27.5	control	136.95	27450.6	0.0050	1
6	SVF#6	19	27.5	PTEN	433.263	28802	0.0150	3.015
7	SVF#6	10	16.5	control	896.477	45455.4	0.0197	1
8	SVF#6	10	16.5	PTEN	37526.342	50902.8	0.7372	37.38
9	SVF#6	14	21	control	25207.38	40361.9	0.6245	1
10	SVF#6	14	21	PTEN	33553.664	34367.8	0.9763	1.563

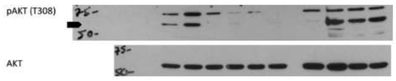

Gel 2

Lane	cell strain	passage	generation	siRNA	AKT	pAKT	pAKT/AKT	Fold
1	SVF#10	18	27	control	25284.451			
2	SVF#10	18	27	PTEN	31423.43			
3	SVF#9	25	29.5	control	20953.945			
4	SVF#9	25	29.5	PTEN	24168.702			
5	SVF#10	16	24.5	control	36576.794	191.192	0.0052	1
6	SVF#10	16	24.5	PTEN	44530.957	23790	0.5342	102.2
7	SVF#10	19	28	control	39983.886	28464.9	0.7119	1
8	SVF#10	19	28	PTEN	19359.48	37363.3	1.9300	2.711

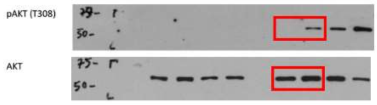

pS6/α-Tubulin

Gel 1

Lane	cell strain	passage	generation	siRNA	α-Tubulin	pS6	pS6/Tubulin	Fold
1	SVF#10	15	17.5	control	29685.765	1591.79	0.0536	1
2	SVF#10	15	17.5	PTEN	25892.258	14296.5	0.5522	10.3
3	SVF#10	19	20.5	control	26662.673	6980.95	0.2618	1
4	SVF#10	19	20.5	PTEN	25048.116	26784.3	1.0693	4.084
5	SVF#10	21	21.5	control	21221.459	985.891	0.0465	1
6	SVF#10	21	21.5	PTEN	24097.409	15372.4	0.6379	13.73

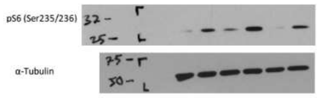

Gel 2

Lane	cell strain	passage	generation	siRNA	α-Tubulin	pS6	pS6/Tubulin	Fold
1	SVF#10	22	31.5	control	25567.078	64222.7	2.5119	1
2	SVF#10	22	31.5	PTEN	22026.501	73514.5	3.3375	1.329
3	SVF#8	6	11.5	control	23997.794	2306.21	0.0961	1
4	SVF#8	6	11.5	PTEN	22733.258	6170.49	0.2714	2.824
5	SVF#6	19	27.5	control	25526.208			
6	SVF#6	19	27.5	PTEN	22336.773			
7	SVF#6	10	16.5	control	32681.501	1563.01	0.0478	1
8	SVF#6	10	16.5	PTEN	31490.723	5657.71	0.1797	3.757
9	SVF#6	14	21	control	23326.752	239.192	0.0103	1
10	SVF#6	14	21	PTEN	27925.693	15612.9	0.5591	54.52

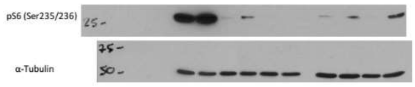

Gel 3

Lane	cell strain	passage	generation	siRNA	α-Tubulin	pS6	pS6/Tubulin	Fold
1	SVF#10	18	27	control	25150.572	471.556	0.0187	1
2	SVF#10	18	27	PTEN	29801.744	24020.1	0.8060	42.99
3	SVF#9	25	29.5	control	28390.179	421.435	0.0148	1
4	SVF#9	25	29.5	PTEN	31141.229	2097.5	0.0674	4.537
5	SVF#10	16	24.5	control	42169.957	5913.85	0.1402	1
6	SVF#10	16	24.5	PTEN	49634.049	29104.5	0.5864	4.181
7	SVF#10	19	28	control	36816.007	41326.6	1.1225	1
8	SVF#10	19	28	PTEN	33632.057	40305.2	1.1984	1.068

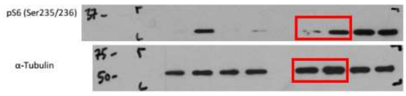

pFOXO1/α-Tubulin

Lane	cell strain	passage	generation	siRNA	α-Tubulin	pFOXO1	pFOXO1/ Tubulin	Fold
1	SVF#6	23	25	control	23465.723	1664.86	0.0709	1
2	SVF#6	23	25	PTEN	25824.551	9051.15	0.3505	4.94
3	SVF#9	16	19.5	control	19014.723	5445.25	0.2864	1
4	SVF#9	16	19.5	PTEN	21137.915	9373.2	0.4434	1.548
5	SVF#10	19	28	control	18774.551	219.778	0.0117	1
6	SVF#10	19	28	PTEN	18489.844	2879.62	0.1557	13.3

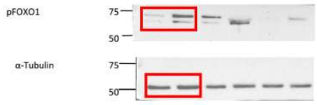

SREBP1/α-Tubulin

Lane	cell strain	passage	generation	siRNA	α-Tubulin	SREBP1	SREBP1/ Tubulin	Fold
1	SVF#10	19	20.5	control	6903.933	9476.12	1.3726	1
2	SVF#10	19	20.5	PTEN	8575.296	19465.1	2.2699	1.654
3	SVF#6	20	19.5	control	5463.64	6880.17	1.2593	1
4	SVF#6	20	19.5	PTEN	5284.004	11169.2	2.1138	1.679
5	SVF#9	12	14.5	control	3411.983	6350.1	1.8611	1
6	SVF#9	12	14.5	PTEN	2969.569	6134.44	2.0658	1.11
7	SVF#8	13	22.5	control	4734.225	3256.18	0.6878	1
8	SVF#8	13	22.5	PTEN	5846.175	6014.9	1.0289	1.496

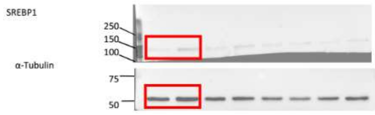

Figure S4: Western blots of PTEN KD/control SVF cells: exposed films and densitometric analyses of PTEN (normalized to α-Tubulin), pAKT (T308) (normalized to total AKT), pS6 (Ser235/236) (normalized to α-Tubulin), NAMPT(normalized to α-Tubulin), p21 (normalized to α-Tubulin), pFOXO1 (normalized to α-Tubulin) and SREBP1 (normalized to α-Tubulin). Western blot images presented in Figures 1(a) and 6(b) were reused (indicated with red boxes).

PTEN CRISPR SVF

PTEN/α-Tubulin

Gel 1

Lane	Cells	guideRNA	α-Tubulin	PTEN	PTEN/Tubulin	Fold
1	CRISPR SVF#1	control	44983.5	35283.2	0.7844	1
2	CRISPR SVF#1	PTEN #3+4	41204.8	23047.4	0.5593	0.7131
3	CRISPR SVF#3	control	39369.1	34844.8	0.8851	1
4	CRISPR SVF#3	PTEN #3+4	32432.8	2196.4	0.0677	0.0765

Gel 2

Lane	Cells	guideRNA	α-Tubulin	PTEN	PTEN/Tubulin	Fold
1	CRISPR SVF#9	control	30284.5	37275.7	1.2309	1
2	CRISPR SVF#9	PTEN #3+4	25771.4	40277.4	1.5629	1.2697

Gel 3

Lane	Cells	guideRNA	GAPDH	PTEN	PTEN/GAPDH	Fold
1	CRISPR SVF#6	control	46613.2	25935.4	0.5564	1
2	CRISPR SVF#6	control	37796.5	20527.1	0.5431	0.9761
3	CRISPR SVF#6	PTEN #3	48217.2	22996.5	0.4769	0.8572
4	CRISPR SVF#6	PTEN #3+4	50484.9	16209.0	0.3211	0.5770

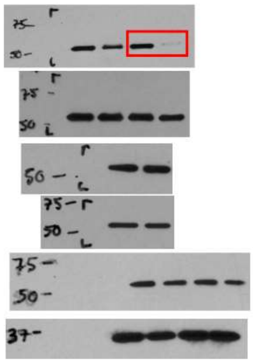

pAKT/AKT

Gel 1

Lane	Cells	guideRNA	pAKT	AKT	pAKT/AKT	Fold
1	CRISPR SVF#1	control	24115.673	47316.22	0.50967032	1
2	CRISPR SVF#1	PTEN #3+4	15713.116	42532.735	0.369435824	0.724852536
3	CRISPR SVF#3	control	16171.53	46697.271	0.346305676	1
4	CRISPR SVF#3	PTEN #3+4	28030.016	43814.614	0.639741252	1.847331118

Gel 2

Lane	Cells	guideRNA	pAKT	AKT	pAKT/AKT	Fold
1	CRISPR SVF#9	control	18924.9	45458.3	0.4163	1
2	CRISPR SVF#9	PTEN #3+4	40323.7	41352.1	0.9751	2.3423

Gel 3

Lane	Cells	guideRNA	pAKT	AKT	pAKT/AKT	Fold
1	CRISPR SVF#6	control	1557.7	34414.0	0.0453	1
2	CRISPR SVF#6	control	1137.2	24325.5	0.0468	1.0329
3	CRISPR SVF#6	PTEN #3	18959.9	32016.1	0.5922	13.0837
4	CRISPR SVF#6	PTEN #3+4	43735.2	37969.8	1.1518	25.4480

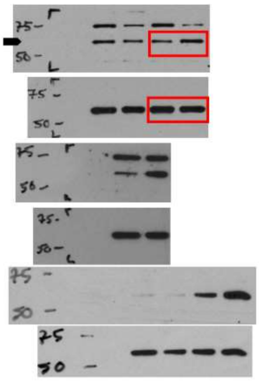

pS6/α-Tubulin

Gel 1

Lane	Cells	guideRNA	α-Tubulin	pS6	pS6/Tubulin	Fold
1	CRISPR SVF#1	control	19917.5	29887.6	1.5006	1
2	CRISPR SVF#1	PTEN #3+4	20513.1	47436.4	2.3125	1.5411
3	CRISPR SVF#3	control	24033.8	20507.5	0.8533	1
4	CRISPR SVF#3	PTEN #3+4	22460.1	49393.8	2.1992	2.5773

Gel 2

Lane	Cells	guideRNA	α-Tubulin	pS6	pS6/Tubulin	Fold
1	CRISPR SVF#9	control	30284.5	527.7	0.0174	1
2	CRISPR SVF#9	PTEN #3+4	25771.4	19182.4	0.7443	42.7126

Gel 3

Lane	Cells	guideRNA	GAPDH	pS6	pS6/GAPDH	Fold
1	CRISPR SVF#6	control	55297.3	8164.4	0.1476	1
2	CRISPR SVF#6	control	41454.0	3526.6	0.0851	0.5762
3	CRISPR SVF#6	PTEN #3	35198.0	23966.1	0.6809	4.6117
4	CRISPR SVF#6	PTEN #3+4	37901.3	49345.4	1.3019	8.8180

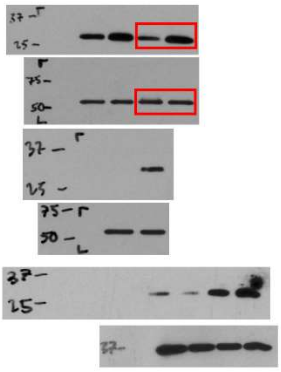

Constitutive active FOXO1 overexpression in CRISPR SVF

FOXO1 active/α-Tubulin

Gel 1

Lane	Cells	guideRNA	Plasmid	α-Tubulin	FOXO1 (FLAG)	FOXO1/Tubulin	Fold
1	CRISPR SVF#6 p16	PTEN #3+4	control	7418.8	476.2	0.0642	1
2	CRISPR SVF#6 p16	PTEN #3+4	FOXO1 active	9221.7	13958.1	1.5136	23.58
3	CRISPR SVF#6 p17	PTEN #3+4	control	7703.0	246.1	0.0320	1
4	CRISPR SVF#6 p17	PTEN #3+4	FOXO1 active	7416.1	7366.7	0.9933	31.09

Gel 2

Lane	Cells	guideRNA	Plasmid	α-Tubulin	FOXO1 (FLAG)	FOXO1/Tubulin	Fold
1	CRISPR SVF#6 p23	PTEN #3+4	control	5765.4	19.0	0.0033	1
2	CRISPR SVF#6 p23	PTEN #3+4	FOXO1 active	6649.6	9061.5	1.3627	414.60

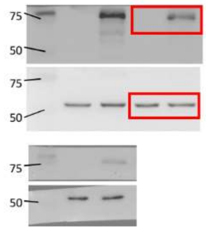

SREBP1/α-Tubulin

Gel 1

Lane	Cells	guideRNA	Plasmid	α-Tubulin	SREBP1	SREBP1/Tubulin	Fold
1	CRISPR SVF#6 p16	PTEN #3+4	control	7418.8	5959.8	0.8033	1
2	CRISPR SVF#6 p16	PTEN #3+4	FOXO1 active	9221.7	3821.9	0.4145	0.52
3	CRISPR SVF#6 p17	PTEN #3+4	control	7703.0	7921.5	1.0284	1
4	CRISPR SVF#6 p17	PTEN #3+4	FOXO1 active	7416.1	5395.6	0.7276	0.71

Gel 2

Lane	Cells	guideRNA	Plasmid	α-Tubulin	SREBP1	SREBP1/Tubulin	Fold
1	CRISPR SVF#6 p23	PTEN #3+4	control	5765.4	3939.5	0.6833	1
2	CRISPR SVF#6 p23	PTEN #3+4	FOXO1 active	6649.6	1095.8	0.1648	0.24

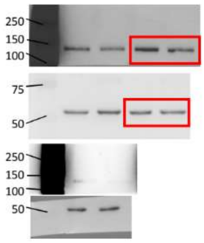

Figure S5: Western blots of PTEN CR/control SVF cells: exposed films and densitometric analyses of PTEN (normalized to α-Tubulin), pAKT (T308) (normalized to total AKT), pS6 (Ser235/236) (normalized to α-Tubulin), FOXO1 (FLAG antibody, normalized to α-Tubulin) and SREBP1 (normalized to α-Tubulin). Western blot images presented in Figures 1(b) and 6(d) were reused (indicated with red boxes).

PTEN KD

NAMPT/α-Tubulin

Gel 1

Lane	cell strain	passage	generation	siRNA	α-Tubulin	NAMPT	NAMPT/Tubulin	Fold
1	SVF#10	15	17.5	control	29685.8	11692.5	0.3939	1
2	SVF#10	15	17.5	PTEN	25892.3	25617.6	0.9894	2.5119
3	SVF#10	19	20.5	control	26662.7	11961.0	0.4486	1
4	SVF#10	19	20.5	PTEN	25048.1	24268.8	0.9689	2.1598
5	SVF#10	21	21.5	control	21221.5	19353.1	0.9120	1
6	SVF#10	21	21.5	PTEN	24097.4	23821.2	0.9885	1.0840

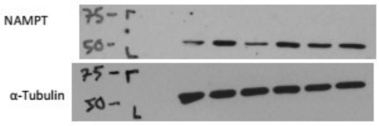

Gel 2

Lane	cell strain	passage	generation	siRNA	α-Tubulin	NAMPT	NAMPT/Tubulin	Fold
1	SVF#10	18	27	control	25150.572	18085.338	0.7191	1
2	SVF#10	18	27	PTEN	29801.744	29915.401	1.0038	1.3960
3	SVF#9	25	29.5	control	28390.179	20071.702	0.7070	1
4	SVF#9	25	29.5	PTEN	31141.229	31966.886	1.0265	1.4519
5	SVF#10	16	24.5	control	42169.957	33930.229	0.8046	1
6	SVF#10	16	24.5	PTEN	49634.049	50054.726	1.0085	1.2534
7	SVF#10	19	28	control	36816.007	37188.907	1.0101	1
8	SVF#10	19	28	PTEN	33632.057	43514.605	1.2938	1.2809

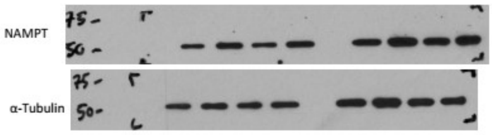

p21/α-Tubulin

Gel 1

Lane	cell strain	passage	generation	siRNA	α-Tubulin	p21	p21/Tubulin	Fold
1	SVF#10	18	27	control	25150.572	12232.974	0.4864	1
2	SVF#10	18	27	PTEN	29801.744	5668.246	0.1902	0.3910
3	SVF#9	25	29.5	control	28390.179	11907.723	0.4194	1
4	SVF#9	25	29.5	PTEN	31141.229	8778.782	0.2819	0.6721
5	SVF#10	16	24.5	control	42169.957	32319.451	0.7664	1
6	SVF#10	16	24.5	PTEN	49634.049	15655.045	0.3154	0.4115
7	SVF#10	19	28	control	36816.007	25134.057	0.6827	1
8	SVF#10	19	28	PTEN	33632.057	4063.104	0.1208	0.1770

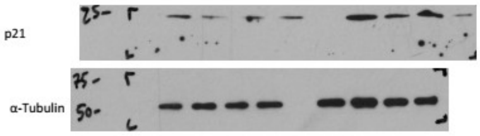

Gel 2

Lane	cell strain	passage	generation	siRNA	α-Tubulin	p21	p21/Tubulin	Fold
1	SVF#10	22	31.5	control	25002.208	16890.459	0.6756	1
2	SVF#10	22	31.5	PTEN	28004.329	7717.246	0.2756	0.4079
3	SVF#8	6	11.5	control	25806.501	17323.752	0.6713	1
4	SVF#8	6	11.5	PTEN	30205.886	7867.246	0.2605	0.3880
5	SVF#6	19	27.5	control	23217.016	15644.974	0.6739	1
6	SVF#6	19	27.5	PTEN	24411.501	2608.912	0.1069	0.1586
7	SVF#6	10	16.5	control	28875.522	4094.447	0.1418	1
8	SVF#6	10	16.5	PTEN	41495.463	97.778	0.0024	0.0166

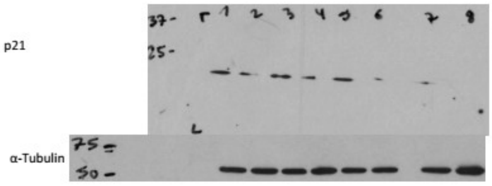

Longterm culture

Gel 1

Lane	cell strain	passage	generation	days	α-Tubulin	PTEN	PTEN/Tubulin	NAMPT	NAMPT/Tubulin	pAkt	Akt	pAKT/AKT
1	SVF#6	4	5.5	11	21897.7	2176.0	0.0994	10473.0	0.4783	56901.7	1123.8	50.6324
2	SVF#6	8	12	24	36210.7	8694.3	0.2401	4683.2	0.1293	42782.3	28394.1	1.5067
3	SVF#6	5	8.5	17	34287.5	2348.0	0.0685	5311.5	0.1549	38720.3	27248.1	1.4210
4	SVF#6	12	17.5	35	44295.3	20148.3	0.4549	8349.9	0.1885	7610.3	41283.3	0.1843
5	SVF#6	14	19.5	39	45090.0	40007.0	0.8873	1639.7	0.0364	16317.2	38784.3	0.4207
6	SVF#6	16	21.5	43	36260.4	29828.0	0.8226	544.0	0.0150	26400.0	33258.7	0.7938
7	SVF#6	18	22.5	45	22367.2	17480.7	0.7815	408.3	0.0183	16106.4	15828.7	1.0175
8	SVF#6	19	23	46	31162.5	22429.2	0.7198	3111.9	0.0999	18106.7	34120.4	0.5307
9	SVF#6	21	26.5	53	38233.7	23050.0	0.6029	2255.5	0.0590	5655.3	32125.4	0.1760
10	SVF#6	21	27	54	36219.0	18207.8	0.5027	968.6	0.0267	4665.3	36954.7	0.1262
11	SVF#6	22	28.5	57	36973.0	27925.5	0.7553	1786.5	0.0483	404.3	45943.9	0.0088
12	SVF#6	20	40.5	81	33873.7	17538.0	0.5177	391.0	0.0115	16532.7	17372.9	0.9516

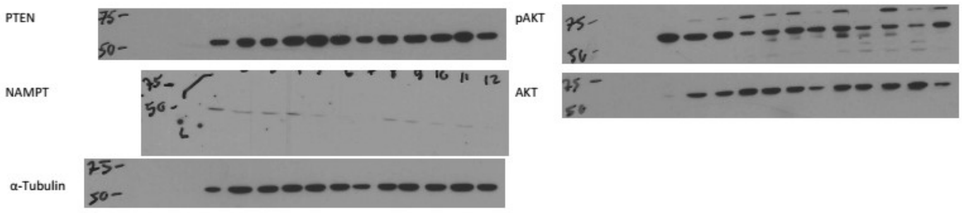

Gel 2

Lane	cell strain	passage	generation	ys in cult	α-Tubulin	PTEN	PTEN/Tubulin	NAMPT	NAMPT/Tubulin	pAkt	Akt	pAKT/AKT
1	SVF#8	2	2	4	14337.7	12774.77	0.890991582	14437	1.006936259	29543.81	3388.518	8.71879978
2	SVF#8	3	3.5	7	24164.33	52807.38	2.185344266	6341.5	0.262433885	41677.32	16287.34	2.55887825
3	SVF#8	5	8.5	17	42892.41	37488.32	0.874008245	558.92	0.013030744	56173.32	2402.326	23.3828881
4	SVF#8	11	18.5	37	84696.81	49400.24	0.583259747	198.95	0.002348967	13984.12	20216.09	0.69173218
5	SVF#8	12	21	42	69102.91	23498.9	0.340056591	777.65	0.011253477	42.536	14095.02	0.0030178
6	SVF#8	13	22.5	45	57271.12	38048.1	0.664350549	277.9	0.004852341	8259.246	11969.27	0.69003757
7	SVF#8	14	25.5	51	50289.697	68391.49	1.359950329	717.75	0.014272287	7757.368	32665.81	0.23747668

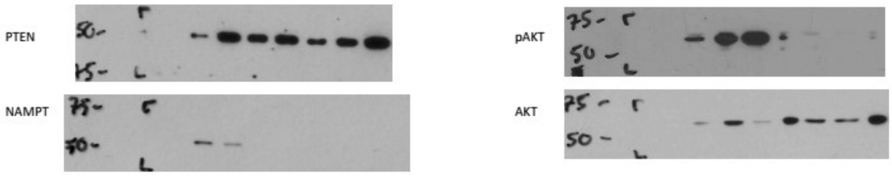

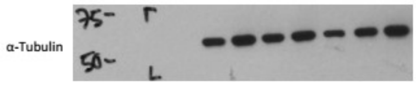

Gel 3

Lane	cell strain	passage	generation	ys in cultı	α-Tubulin	PTEN	PTEN/Tubulin	NAMPT	NAMPT/Tubulin	pAkt	Akt	pAKT/AKT
1	SVF#9	1	1	2	14337.7	76.778	0.005354973	61101	4.261530789	7211.966	2503.69	2.88053473
2	SVF#9	1	1.5	3	24164.33	14126.92	0.584618733	90193	3.732468063	17232.77	13080.48	1.31744171
3	SVF#9	6	8.5	17	42892.41	39505.61	0.921039643	47385	1.104740909	77.607	38965.78	0.00199167
4	SVF#9	9	13.5	27	84696.81	65594.47	0.774462108	32540	0.384194281	137.728	69093.95	0.00199334
5	SVF#9	12	14.5	29	69102.91	62116.41	0.898897167	18288	0.264652096	29267.32	64161.54	0.45615052
6	SVF#9	13	16.5	33	57271.12	20776.06	0.362766784	10323	0.180255598	9554.147	42402.8	0.22531878
7	SVF#9	18	24	48	44063.59	25981.66	0.589640109	18145	0.411791005	73.192	48147.8	0.00152015

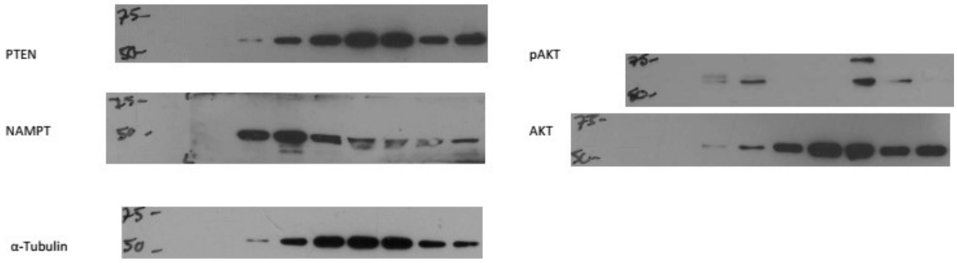

Gel 4

Lane	cell strain	passage	generation	ys in cultı	Tubulin	PTEN	PTEN/Tubulin	NAMPT	NAMPT/Tubulin	pAkt	Akt	pAKT/AKT
1	SVF#10	1	3	6	22996.19	9064.288	0.394164773	54166	2.355448446	267.021	36170.77	0.00738223
2	SVF#10	2	4.5	9	25914.02	20824.28	0.803591261	15888	0.613089748	29203.35	38533.37	0.75787168
3	SVF#10	3	5.5	11	29100.14	24330.42	0.836092885	7606.6	0.261394997	12482.07	44235.22	0.28217493
4	SVF#10	6	10	20	21255.58	29670.3	1.395882869	14390	0.677006697	36794.81	37088.32	0.99208619
5	SVF#10	9	14.5	29	40137.3	66097.88	1.646794378	2711.4	0.067553049	485.435	72107.16	0.00673213

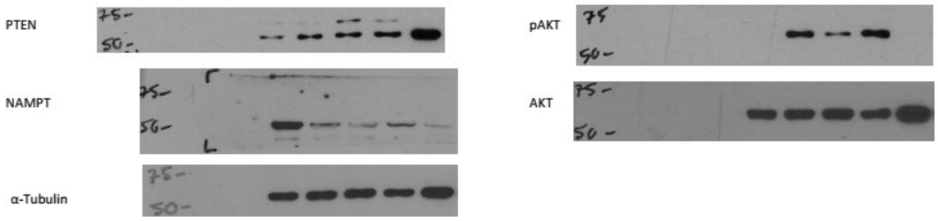

Figure S6: Western blots of four SVF cell long term cultures: exposed films and densitometric analyses of PTEN (normalized to α-Tubulin), pAKT (T308) (normalized to total AKT) and pS6 (Ser235/236) (normalized to α-Tubulin).

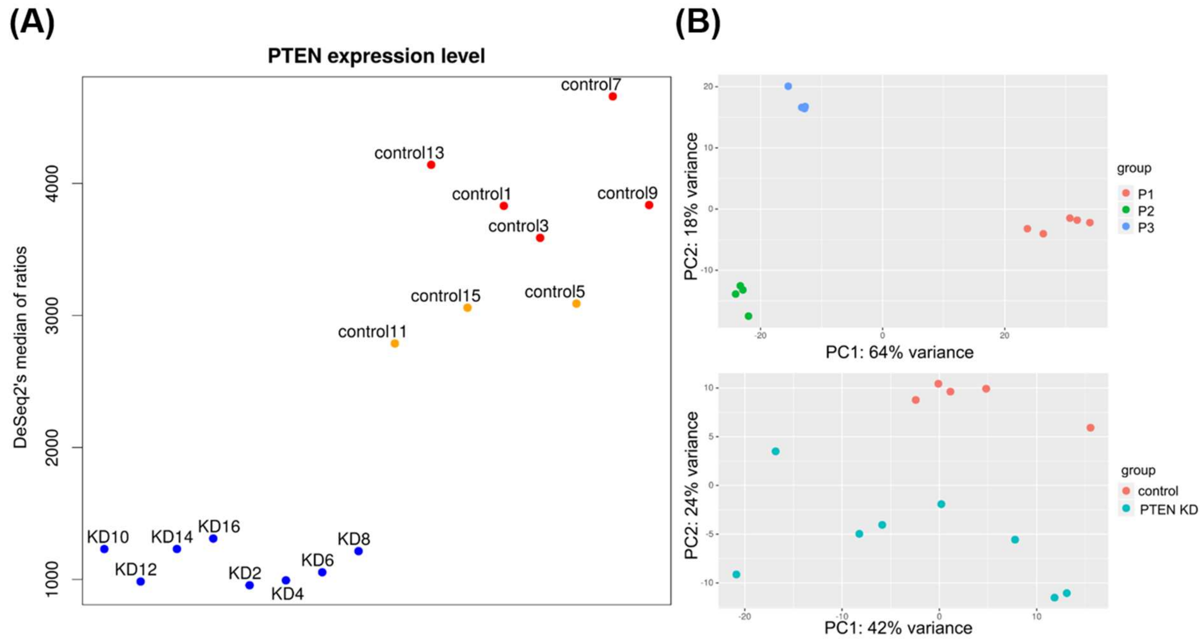

Figure S7: (A) Normalized read counts mapping to *PTEN* mRNA in the investigated samples. (B) PCA of samples clearly cluster according patients (upper panel), after removing the variance caused by the individual cell donors the second principal component (PC2) segregates the samples into control *vs*. PTEN (lower panel).

Supporting information tables:

Lists of differentially expressed genes (Supporting information Tables S1-3) are available separately.

Table S4. SVF cell cultures.

Name	Sex	Age at resection	Origin
SVF#1	female	31	visceral
SVF#3	female	30	visceral
SVF#5	female	33	visceral
SVF#6	male	27	visceral
SVF#8	male	37	visceral
SVF#9	female	32	visceral
SVF#10	female	29	visceral
SVF#11S	male	18	subcutaneous
SVF#12S	male	24	subcutaneous
SVF#14S	female	28	subcutaneous

Table S5. Antibodies used for flow cytometry.

Marker	Fluorophore	Distributor	Clone	Cat. no
MSCA1	PE	Miltenyi	W8B2	130-093-587
CD34	FITC	Miltenyi	AC136	130-113-178
CD14	PeVio770	Miltenyi	Tük4	130-113-149
CD271	APC	Miltenyi	ME20.4-1-H4	130-113-418
CD8	PerCP	BD Horizon	SK1	345774
CD31	V450	BD Horizon	WM59	561653
CD45	BV510	BioLegend	HI30	304036

Table S6. Flow cytometry analysis of cell surface markers.

cells	passage	generation	single cells	viable cells	CD8+	CD14+	CD31+	CD34+	CD45+	CD271+	MSCA1+
LipPD1	6	8	97%	77.60%	none	none	none	none	none	4.70%	4.74%
LipPD1	28	34	93.60%	66.70%	none	6%	none	3.62%	none	3.86%	1.51%
SVF#1	6	19	95.30%	57.00%	none	2.56%	none	18.60%	none	3.67%	none
SVF#1	12	17.5	93.70%	51.90%	none	2.76%	none	22.00%	none	1.58%	3.00%
SVF#1	22	35.5	99.10%	71.20%	none	none	none	29.00%	none	none	1.63%
SVF#5	0	0	98.90%	23.00%	none	9.10%	3.04%	58.70%	6.18%	15.80%	5.96%
SVF#5	11	25.5	96.50%	79.90%	none	none	none	2%	none	none	none
SVF#6	0	1.5	99.60%	8.04%	none	2.98%	2%	none	none	19%	19%
SVF#6	16	19	96.20%	79.30%	none	none	none	12.90%	none	none	4.46%
SVF#6	23	31	97.10%	74.80%	none	none	none	38.60%	none	none	2.44%
SVF#8	0	1	99.20%	53.10%	1.59%	2.82%	3.53%	74.40%	2.67%	55.60%	7.29%
SVF#8	11	23.5	92%	65.50%	none	none	none	17%	none	none	none
SVF#9	0	1	99%	33.90%	5.30%	9.44%	2.72%	56.70%	4.50%	27.20%	5.17%
SVF#9	4	6	95.50%	74.50%	none	none	none	none	none	23.20%	11.60%
SVF#9	14	20	97.30%	82.60%	none	none	none	44.70%	none	none	1.11%
SVF#10	15	20.5	96.90%	89.20%	none	none	none	5.94%	none	2.27%	1.54%
SVF#10	15	24	94.50%	77.90%	none	none	none	15%	none	none	20.20%
SVF#10	23	27	90.40%	79%	none	none	none	none	none	none	none
SVF#11S	22	29	95.30%	65.40%	none	none	none	6.18%	none	15.60%	none
SVF#12S	18	26.5	96%	78%	none	none	none	12.80%	none	none	none
SVF#14S	8	12.5	96.40%	87%	none	none	none	9.53%	none	1.72%	none
SVF #3	0	3.5	99%	26%	none	none	none	5.14%	none	1.62%	2.75%

Table S7. crRNAs used for CRISPR/Cas9 *PTEN* knockout.

#	Design ID	crRNA Sequence	PAM	Strand
1	Hs.Cas9.PTEN.1.AC	TTATCCAAACATTATTGCTA	TGG	+
2	Hs.Cas9.PTEN.1.AF	TATCCAAACATTATTGCTAT	GGG	+
3	CD.Cas9.VCTN3398.AH	ATATCTGAGTACTTTAGTTA	AGG	-
4	CD.Cas9.VCTN3398.BE	TTTCCTGCAGAAAGACTTGA	AGG	+

Table S8. Antibodies used for Western blot (Wb) and immunofluorescence staining (IF).

Primary antibody	Dilution	Distributor	Cat. no
PTEN (138G6) Rabbit map	1:1000 TBS-T 5%BSA (Wb)	CST	#9559
AKT antibody Rabbit polyclonal Ab	1:1000 TBS-T 5%BSA (Wb)	CST	#9272
Phospho-AKT (Thr308) (224F9) Rabbit mAb	1:1000 TBS-T 5%BSA (Wb)	CST	#4056
Phospho-S6 Ribosomal protein (Ser235/236) (D57.2.2E) XP® Rabbit mAb	1:1000 TBS-T 5%BSA (Wb)	CST	#4858
mAb anti -NAMPT Antibody	1:1000 TBS-T 5%BSA (Wb)	Novus Biologicals	#NBP1-96585
P21 Waf 1/Cip1 D(S60)	1:1000 TBS-T 5%BSA (Wb)	CST	#2496
alpha Tublin (11H10) Rabbit mAb	1:2000 TBS-T 5%BSA (Wb)	CST	#2125
Phospho-FoxO1 (Ser256) Antibody	1:1000 TBS-T 5%BSA (Wb)	CST	#9461
SREBP-1 polyclonal antibody	1:1000 TBS-T 5% milk (Wb)	Santa Cruz	sc-8984
ANTI-FLAG Rabbit antibody	1:1000 TBS-T 5%BSA (Wb)	Sigma	F7425
KI-67 (MIB-1) Mouse mAb	1:200 IF-buffer (IF)	Dako	P0447
**Secondary antibody**	**Dilution**	**Supplier**	**Cat. no**
Polyclonal goat anti-rabbit immunoglobulin/HRP	1:2000 TBS-T 5% milk (Wb)	Dako	P0447
Polyclonal goat anti-mouse immunoglobulin/HRP	1:2000 TBS-T 5% milk (Wb)	Dako	P0448
Alexa Fluor 488 goat anti-mouse IgG H+L	1:1000 IF-buffer (IF)	Invitrogen	A11001

Table S9. Primers used for RT-qPCR.

Gene	Forward	Reverse	Probe
HPRT	GGCAGTATAATCCAAAGATGGTCAA	GTCTGGCTTATATCCAACACTTCGT	CAAGCTTGCTGGTGAAAAGGACCCC
TBP	TTGTAAACTTGACCTAAAGACCATTGC	TTCGTGGCTCTCTTATCCTCATG	AACGCCGAATATAATCCCAAGCGGTTTG
PTEN	TGTAAAGCTGGAAAGGGACGA	GGAATAGTTACTCCCTTTTTGTCTC	
ADIPOQ	GGCCGTGATGGCAGAGAT	CCTTCAGCCCCGGGTACT	CGATGTCTCCCTTAGGACCAATAAGACCTGG
FABP4	GCTTTTGTAGGTACCTGGAAACTTG	ACACTGATGATCATGTTAGGTTTGG	CCTGGTGGCAAAGCCCACTCCTCAT
FASN	GGCAAATTCGACCTTTCTCAGA	GGACCCCGTGGAATGTCA	CACCCGCTCGGCATGGCTATCTT
CDKN1A (*p21*)	CGAAGTCAGTTCCTTGTGGAG	CATGGGTTCTGACGGACAT	
CDKN2A (*p16*)	CTTCGGCTGACTGGCTGG	TCATCATGACCTGGATCGGC	
CDKN2B (*p15*)	AGGCGCGCGATCCAG	GGTGAGAGTGGCAGGGTCT	
HIPK2	GCTCAAGATGGCAGATTCCG	ACTTGCACATGTGAGGCCATA	
FOXO1	CCTACGCCGACCTCATCAC	AATTGAATTCTTCCAGCCCGC	
RNF144B	TGCCTGAAACAGTACATGCAG	ACCAAACAGGCAATCTCAGC	
SREBF-1	ACCGACATCGAAGGTGAAGT	CAGGGAAGTCACTGTCTTGGT	
